# Nut consumption and risk of metabolic syndrome and overweight/obesity: a meta-analysis of prospective cohort studies and randomized trials

**DOI:** 10.1186/s12986-018-0282-y

**Published:** 2018-06-22

**Authors:** Hang Li, Xia Li, Sheng Yuan, Yalei Jin, Jinping Lu

**Affiliations:** 1 Department of Cardiology, Zhongnan Hospital of Wuhan University, Wuhan University, Wuhan, 430071 China; 2 Department of Geratology, Zhongnan Hospital of Wuhan University, Wuhan University, Wuhan, 430071 China

**Keywords:** Nutrition, Metabolic syndrome, Overweight, Obesity

## Abstract

**Background:**

Nut consumption has been shown to reduce the risk of cardiovascular disease. However, its role in the prevention of metabolic disorders, such as metabolic syndrome (Mets) and overweight/obesity, remains controversial. We therefore conducted a meta-analysis to determine the association of nut consumption with Mets and overweight/obesity.

**Methods:**

Eligible studies were identified by searching the PubMed and Embase databases and by reviewing the references of relevant literatures. We used random effect models to pool the studies-specific risk ratio (RR) and weighted mean difference (WMD).

**Results:**

This meta-analysis included six prospective cohort studies with 420,890 subjects and 62 randomized feeding trials with 7184 participants. Among the cohort studies, the summary RR for every 1-serving/week increase in nut intake was 0.96 (95% confidence interval [CI]: 0.92 to 0.99; *n* = 3) for Mets, 0.97 (95% CI: 0.95 to 0.98; *n* = 2) for overweight/obesity, and 0.95 (95% CI: 0.89 to 1.02; n = 2) for obesity. Pooling of randomized trials indicated that nut consumption was related to a significant reduction in body weight (WMD: − 0.22 Kg, 95% CI: -0.40 to − 0.04), body mass index (WMD: − 0.16 Kg/m^2^, 95% CI: -0.31 to − 0.01), and waist circumference (WMD: − 0.51 cm, 95% CI: -0.95 to − 0.07). These findings remained stable in the sensitivity analysis, and no publication bias was detected.

**Conclusion:**

Nut consumption may be beneficial in the prevention of Mets and overweight/obesity. Additional prospective studies are needed to enhance these findings and to explore the metabolic benefits for specific subclasses of nut.

**Electronic supplementary material:**

The online version of this article (10.1186/s12986-018-0282-y) contains supplementary material, which is available to authorized users.

## Background

Metabolic syndrome (MetS) is recognized as a cluster of interrelated metabolic abnormalities, including central adiposity, hypertension, hyperglycemia, and dyslipidemia [1]. It has been estimated that nearly 20–30% of the global adult population are suffering from Mets [2]; and in the U.S., the overall prevalence is 33% [3]. Patients with Mets have greater risks of developing cardiovascular disease, type 2 diabetes mellitus and common cancers [4, 5]. Overweight/obesity is also a widespread burdensome disease with severe health consequence. In the year 2016, the World Health Organization reported that more than 1.9 billion people aged 18 years or older worldwide were overweight, and over 650 million adults were obese [6]. Therefore, reducing the prevalence of these metabolic disorders is imperative to improve human health and to ameliorate the related social and economical burdens.

In recent decades, lifestyle modifications, in particular a healthy diet, have been increasingly considered to play critical roles in the prevention of metabolic diseases. Previous reports have suggested that dietary intake of fruits and vegetables, dairy products, and coffee may reduce the risk of Mets or overweight/obesity [7–10], while high consumption of red and processed meat increases this risk [11, 12]. Nuts are rich in bioactive nutrients such as healthy fats, dietary fiber, phytosterols, vitamins, and minerals, which are known to have salutary effects on cardiometabolic health [13]. For habitual nut intake, epidemiological studies have confirmed its preventive role against cardiovascular disease, type 2 diabetes mellitus, and cancers [14]. Although emerging evidence from clinical trials have also shown that nut supplementation could favorably affect metabolic features and body weight management [15, 16], whether nut consumption is associated with the overall risks of Mets and overweight/obesity remains in debate. This may be attributed to the fact that there are limited studies investigating these associations, and they yielded inconsistent results. Some of them showed that nut consumption was inversely correlated with risk of MetS or overweight/obesity [17–20], while others demonstrated null associations [21, 22]. Herein, we performed a meta-analysis of pertinent cohort studies to determine the role of nut intake in preventing Mets and overweight/obesity. To improve the reliability of the findings, the data of anthropometrical outcomes in randomized feeding trials were also pooled, including changes in body weight, body mass index (BMI), and waist circumference.

## Methods

### Search strategy

This work was implemented in accordance with the Meta-analysis Of Observational Studies in Epidemiology guidelines [23]. We identified eligible studies by systematically searching the PubMed and Embase databases from inception to December 2017, utilizing the free-text word and Medical Subject Headings terms as follows: (“nut” OR “walnut” OR “peanut” OR “hazelnut” OR “almond” OR “pistachio” OR “cashew” OR “macadamia” OR “pecan” OR “pine nut” OR “brazil nut”) AND (“metabolic syndrome” OR “Mets” OR “overweight” OR “weight gain” OR “obesity” OR “obese” OR “adiposity” OR “adipose” OR “body weight” OR “body mass index” OR “BMI” OR “waist circumstance” OR “hypertension” OR “blood pressure” OR “hypercholesterolemia” OR “dyslipidemia” OR “cholesterol” OR “triglycerides” OR “diabetes mellitus” OR “glucose” OR “glycemia”). Detailed search strategies were listed in Additional file 1: Table S1. Moreover, the references of relevant literature were manually scrutinized to find potential complements. Only full-text studies published in English were considered.

### Inclusion criteria

To be included in this meta-analysis, the cohort studies should meet the following conditions: 1) the exposure of interest was nut consumption; 2) the outcomes of interest included incidence of Mets or overweight/obesity; 3) the adjusted risk estimates, such as relative risks (RRs), were provided for at least three quantitative categories of nut consumption. Alternatively, studies that reported the risk estimates for every 1-serving/week increment in nut consumption were also eligible; 5) the median or mean consumption (or cut-off values) and number of cases were available for each category of nut intake. We also included randomized feeding trials that examined the effects of a nut-enriched diet on anthropometrical outcomes, including body weight, BMI, and waist circumference. Animal studies, reviews, editorials, and studies of children or adolescents were excluded. When studies pertained to overlapping populations, only the report with the longest follow-up was retained.

### Data collection and quality evaluation

Two reviewers (H.L. and X.L.) independently abstracted the study details, including study author, publication year, study design, country, baseline age, and sample size. For cohort studies, the number of cases, measurement of nut intake, outcomes evaluated, confounders adjusted in multivariable models, and the maximally-adjusted risk estimates of Mets or overweight/obesity for each category of nut intake were additionally recorded. For clinical trials, we also extracted the type and amount of nuts consumed, details of comparative diet, and intervention duration. If necessary, the corresponding author of the original study was contacted for missing data. The methodological quality was appraised by using the Newcastle-Ottawa Scale [24] for cohort studies and the CONsolidated Standards Of Reporting Trials statement [25] for clinical randomized trials. Any divergence between the two reviewers was resolved by discussion with a third reviewer (Y.J.).

### Statistical analyses

In the meta-analysis of cohort studies, RRs and its 95% confidence intervals (CIs) were used to report the summary risk estimates for Mets or overweight/obesity. Because the cut-off points for intake categories were varied among the studies, we calculated an RR for an increase of one serving (equals to 30 g of nuts) per week in nut consumption for each report. The median or mean intake level of nut in each category was defined as the corresponding dose. If the mean or median intake level was not reported, the midpoint of the upper and lower boundaries of each category was used instead. If the highest category was open-ended, we assumed the median level as 1.5-times the lower boundary; and, when the lower boundary of the lowest category was unavailable, we set it to be zero. The method described by Greenland and Longnecker [26] and Orsini et al. [27] was used to compute the trend from the correlated estimates for log RR across categories of nut consumption.

In the meta-analysis of randomized feeding trials, the summarized estimates of changes in body weight, BMI, or waist circumference were presented as weighted mean differences (WMDs). To enable the pooling of data, standard errors or CIs were converted to standard deviations. All anthropometrical data were evaluated at the longest follow-up time according to the intention-to-treat analysis.

The heterogeneity between studies were explored by using the Cochrane Q test (significant level: *p* < 0.1). We also quantified the heterogeneity by the *I*^*2*^ statistic, with adoption of the following cut-off values: little or no heterogeneity (< 25%), moderate heterogeneity (25–75%), and substantial heterogeneity (> 75%). The study-specific estimates were aggregated using a random effect model. Subgroup analyses were conducted according to diagnostic criteria of outcomes (for Mets only), with between-subsets difference confirmed by the Altman and Bland test [28]. To assess the robustness of results from clinical trials, we also performed a sensitivity analysis by omitting study one at a time. Potential publication bias was appraised by Egger’s test. All data analyses were realized using R 3.4.2 (The R Foundation for Statistical Computing, Vienna, Austria) and STATA 13.0 (StataCorp, College Station, TX) software, and *p* values < 0.05 were considered as significant.

## Results

### Search process and characteristics of studies

**Fig. 1:**
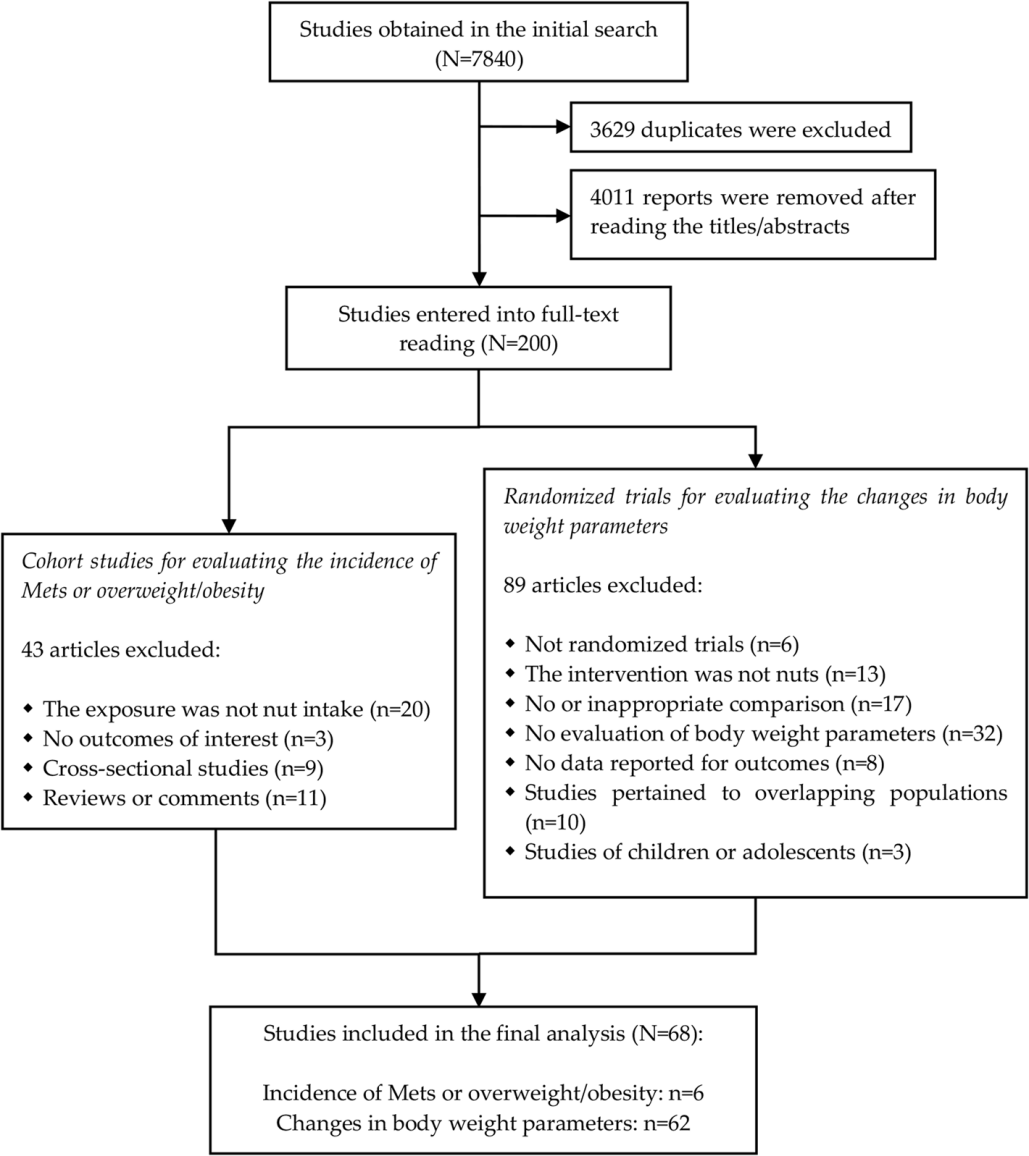
Flow diagram of study search

We obtained 7840 reports in the preliminary search, of which 3629 duplicates and 4011 irrelevant articles were removed. After full-text screening of the retained studies, 132 records were excluded because they failed to meet the eligibility criteria (see the detailed reasons for exclusion in Additional file 1: Table S2). As a result, 68 publications including six prospective cohort studies [17–22] and 62 clinical feeding trials [15, 16, 29–88] were included in this meta-analysis (Fig. 1).

Among the cohort studies, there were 420,890 participants with 4625 Mets cases and 47,235 overweight/obesity cases (Table 1). All studies assessed the nut intake using food-frequency questionnaires. Mets was ascertained by the harmonized definition [1] in one study and by the AHA/NHLBI diagnostic criteria [89] in the other two studies, and overweight/obesity was consistently confirmed by BMI. The most commonly adjusted factors were age, sex, smoking, alcohol intake, physical activity, and total calories intake. In the clinical trials, a total of 7184 subjects were documented, with follow-up duration ranging from 3 to 336 weeks. Other characteristics were shown in Additional file 1: Table S3.

**Table 1:** Characteristics of the included studies

Study	Country	Age (yrs)	Sample size	Cases	Outcomes	Measurement of nuts	Adjustments	NOS score
Bes-Rastrollo 2007 [21]	Spain	38 (mean)	6300	434	Overweight/obesity	FFQ	Age, sex, baseline BMI, leisure time PA, smoking, snacking and television watching	8
Bes-Rastrollo 2009 [17]	US	20–45	51,188	5924	Obesity	FFQ	Age, alcohol intake, PA, smoking, postmenopausal hormone use, oral contraceptive use, baseline BMI, glycemic load, changes in the adherence of prudent and Western dietary patterns and food groups	8
Fernández-Montero 2013 [18]	Spain	38 (mean)	9887	567	Mets	FFQ	Age, BMI, smoking, PA, alcohol intake and total energy intake	8
Freisling 2017 [19]	10 European countries	25–70	197,291	28,244	Overweight/obesity	FFQ	Age, sex, country, baseline BMI, follow-up time in years, total energy intake, educational level, PA, smoking, plausibility of dietary energy reporting and modified relative Mediterranean diet score	
			127,445	12,633	Obesity			8
Hosseinpour-Niazi 2017 [20]	Iran	19–74	1265	276	Mets	FFQ	Age, sex, BMI, smoking, family history of diabetes, PA, fasting glucose, HDL-C, total energy intake, total fiber, percent of protein, percent of carbohydrates, percent of total fat, cholesterol intake, fruit, vegetables and dairy products	7
Lutsey 2008 [22]	US	45–64	9514	3782	Mets	FFQ	Age, sex, race, education, center, total calories, smoking, pack-years, PA and intakes of meat, dairy, fruits, vegetables, whole grains and refined grains	9

BMI, body mass index; FFQ, food-frequency questionnaire; HDL-C, high density lipoprotein cholesterol; Mets, metabolic syndrome; NOS, Newcastle-Ottawa Scale; PA, physical activity

### Nut consumption and risk of Mets

**Fig. 2:**
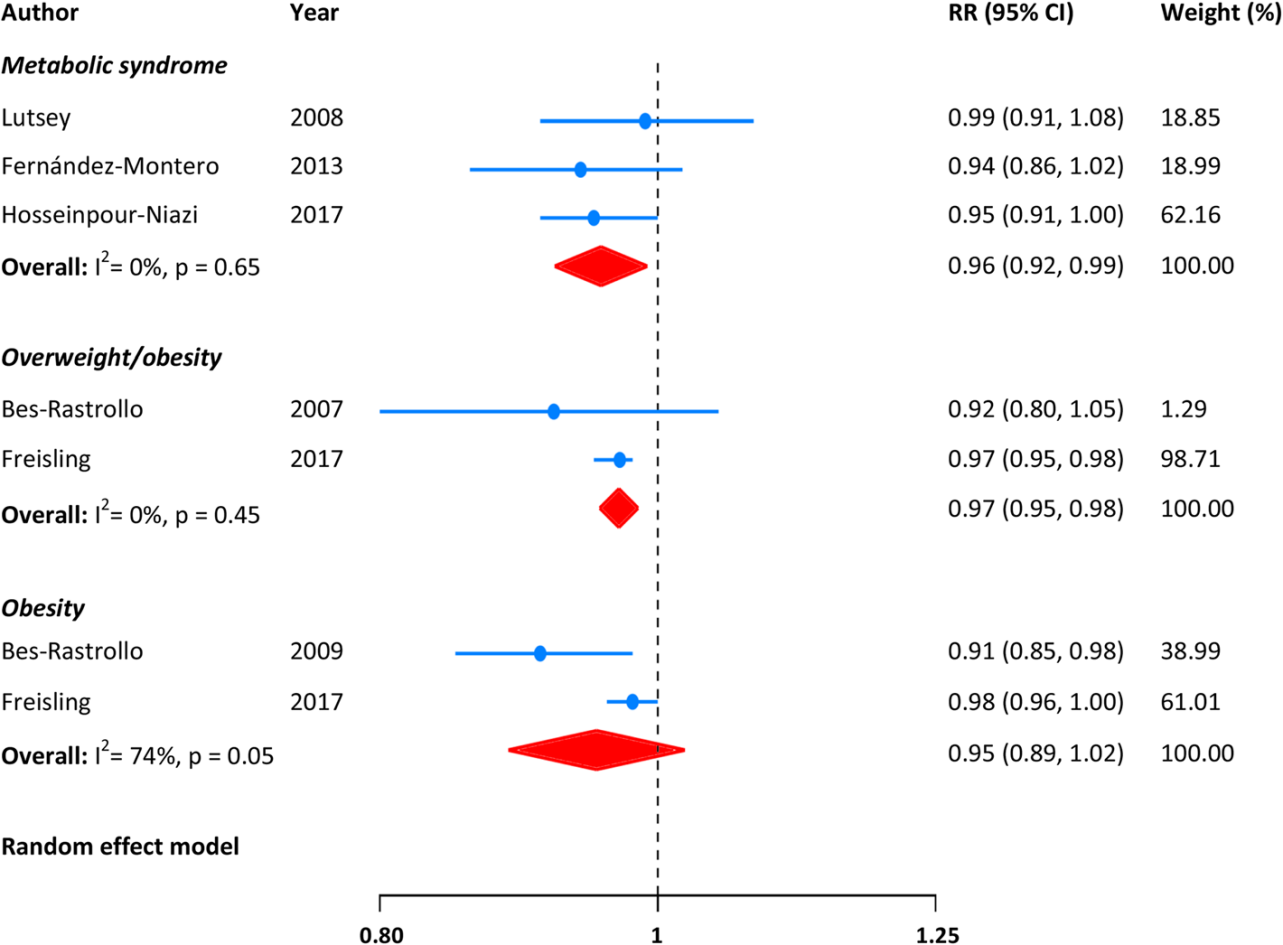
Meta-analysis of nut consumption (per 1-serving increase/week) and risk of Mets and overweight/obesity

Three studies [18, 20, 22] reported the correlation relating nut consumption and risk of Mets, with no statistical heterogeneity across them (I^2^ = 0%, *p* = 0.65). The summary RR of Mets for an increase of 1-serving/week in nut intake was 0.96 (95% CI: 0.92 to 0.99; Fig. 2). The benefits of nut consumption in prevention of Mets may be also existed among studies using the harmonized definition (RR: 0.95, 95% CI: 0.91 to 1.00) or the AHA/NHLBI diagnostic criteria (RR: 0.96, 95% CI: 0.91 to 1.02; p for interaction = 0.78). There was no indication of publication bias from Egger’s test (*p* = 0.72).

### Nut consumption and risk of overweight/obesity

Three studies [17, 19, 21] reported the risk of overweight and/or obesity associated with nut intake. The pooled RR (95% CI) of overweight/obesity for each 1-serving/week increment in nut consumption was 0.97 (0.95 to 0.98), with no heterogeneity across the studies (I^2^ = 0%, *p* = 0.45; Fig. 2). Likewise, nut intake may be also related to a reduced risk of becoming obesity (RR: 0.95, 95% CI: 0.89 to 1.02; I^2^ = 74%, *p* = 0.05; Fig. 2). Evaluation of publication bias was infeasible due to the quite limited number of studies.

### Nut consumption and changes in body weight parameters

**Fig. 3:**
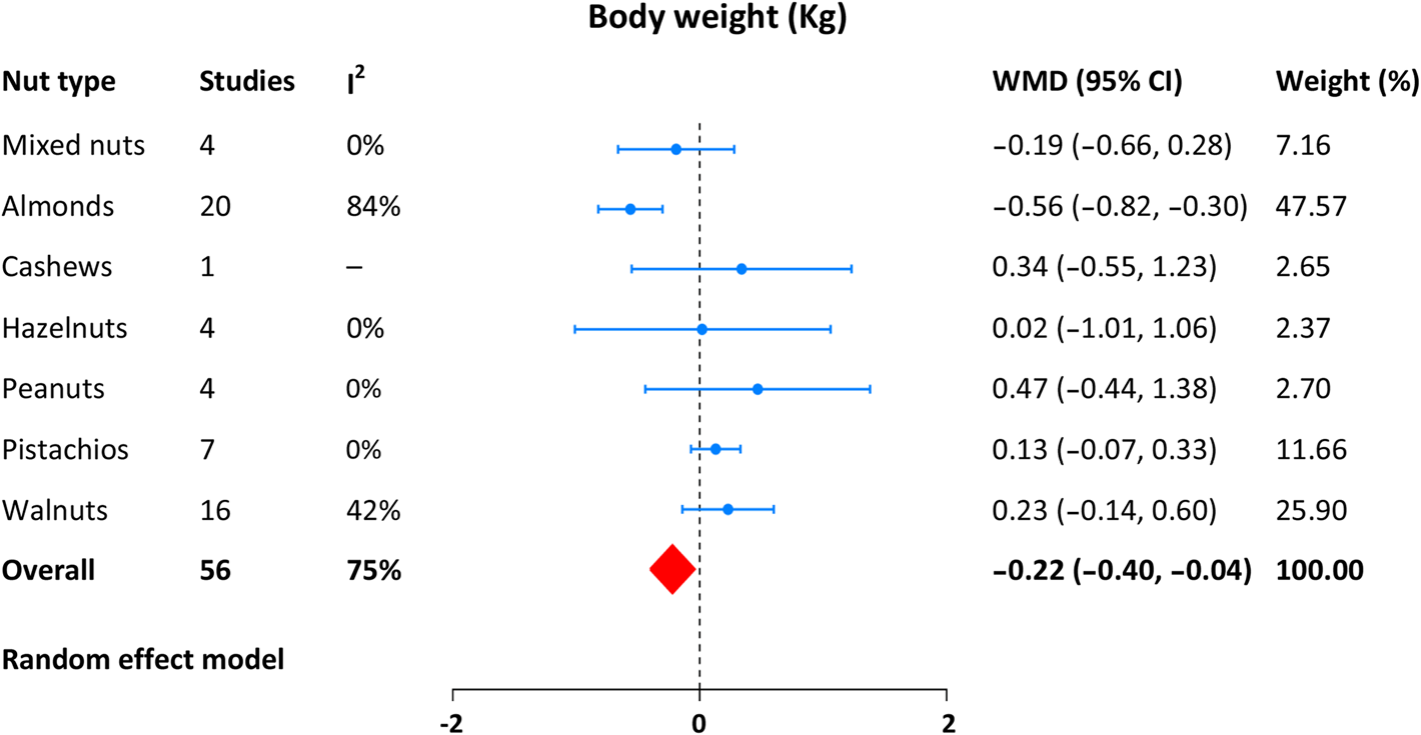
Meta-analysis of nut supplementation and changes in body weight

**Fig. 4:**
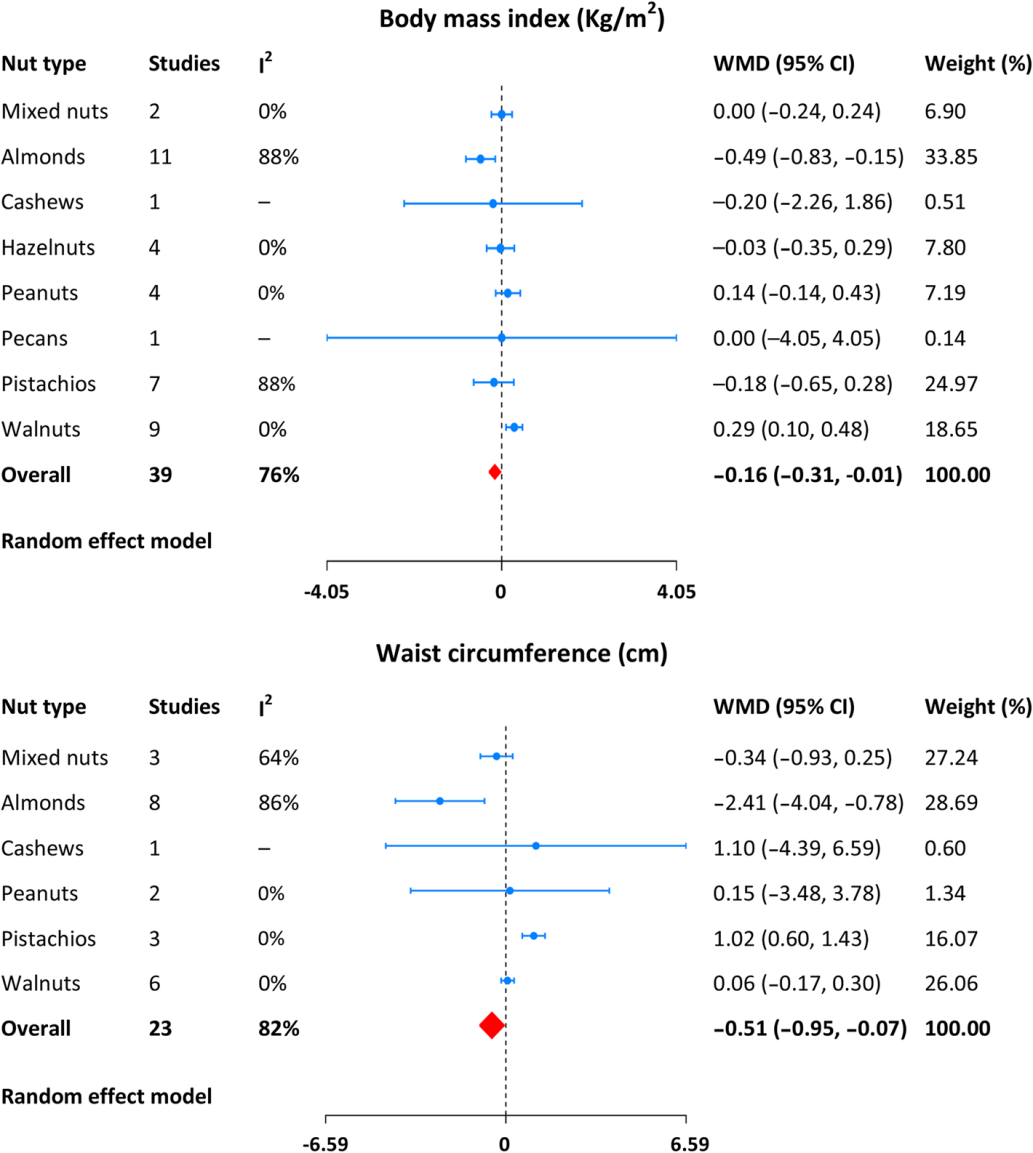
Meta-analysis of nut supplementation and changes in body mass index and waist circumference

The data of body weight, BMI, and waist circumference were provided in 56, 39, and 23 randomized feeding trials, respectively, with substantial heterogeneity across them. Compared with control diet, a nut-enriched diet was associated with a significant decrease in body weight (WMD: − 0.22 Kg, 95% CI: -0.44 to − 0.04; Fig. 3), BMI (WMD: -0.16 Kg/m^2^, 95% CI: -0.31 to − 0.01; Fig. 4), and waist circumference (WMD: − 0.51 cm, 95% CI: -0.95 to − 0.07; Fig. 4). Exclusion of each study in sequence had no significant influence on the pooled results. Egger’s test indicated no potential of publication bias (body weight: *p* = 0.52; BMI: *p* = 0.37; waist circumference: *p* = 18). The detailed results from the individual studies were displayed in Additional file 1: Figures S1–S3.

## Discussion

Nut consumption has been recommended as an important component of healthy diet by the American Heart Association since 2000 [90], but most of the relevant epidemiological studies are to date focused on its protective role against cardiovascular diseases. The present meta-analysis of prospective cohort studies showed that for every 1-serving/week increase in nut consumption, the risk was reduced by 4% for Mets, by 3% for overweight/obesity, and by 5% for obesity only. In addition, the pooled data of randomized feeding trials suggested that nut supplementation could lower body weight, BMI, and waist circumference, further supporting the metabolic benefits of nuts.

Previous reports have suggested a protective role of nut-rich diet against the development of Mets or overweight/obesity. In a meta-analysis of 50 studies totaling 534,906 individuals, Kastorini et al. demonstrated that adherence to the Mediterranean diet enriched with nuts conferred a lower risk of Mets (log hazard ratio: -0.69, 95% CI: -1.24 to − 1.16) and improved waist circumference (− 0.42 cm, 95% CI: -0.82 to − 0.02) [91]. Similarly, a recent nationwide survey of Chilean adults found an inverse correlation between Mediterranean diet score and the prevalence of Mets and overweight or obesity [92]. For dietary intake of nut, accumulating evidence from feeding trials shows that it can reduce blood pressure, improve lipid profiles, and regulate glycemia metabolism and insulin sensitivity [15, 93]. In prospective cohort studies, nut consumption has also been identified to be correlated with decreased weight gain [17, 21]. All of these findings pointed towards the potentially preventive effects of nuts on Mets and overweight/obesity.

There are some possible explanations for the observed benefits of nuts. As a nutritious food, high intake of nuts provides abundant bioactive compounds including unsaturated fatty acids, dietary fiber, plant protein, antioxidants, vitamin E, arginine, phytosterols, and minerals like potassium, calcium, and magnesium. These healthy nutrients alone or in combination may improve inflammatory response, oxidative stress, and endothelial function, thus contributing to the improvements in individual Mets component [94]. Recent viewpoints have also highlighted the importance of gut microbiota in the pathophysiological process of metabolic disease and obesity [95]. There is a notion that the non-bioaccessible substances from nuts (polymerized polyphenols, polysaccharides, and fiber) have prebiotics properties, which serve as substrates for human gut microbiome and cause favorable alterations in microbiome structure [96]. Hence, the health benefits of nut may be, as least in part, explained by its prebiotic effects on gut microbiome. The weight-loss effects of nuts were also likely to be related to the enhanced satiety, increased resting energy expenditure and diet-induced thermogenesis, and incomplete mastication and fat malabsorption [97, 98]. In addition, it deserves to be noted that subjects consuming higher amounts of nut tend to eat less red and processed meat [98]. Such a substitution appears to be helpful for the prevention of Mets and overweight/obesity because intakes of red and processed meat have been suggested to increase the risk of these metabolic abnormalities, as already mentioned above [11, 12].

In this meta-analysis, the inverse association of nut consumption with overweight and/or obesity seems to be counterintuitive, since nuts are a group of foods with high fats and dense calories. However, a previous pooled analysis of controlled feeding trials has shown borderline significant benefits of nut supplementation in the management of body weight, body mass index, and waist circumference [99]. Also, in a recent clinical trial with 6-month interventions, a walnut-enriched reduced-energy diet was found to promote weight loss, which is comparable to a standard reduced-energy-density diet in the context of a behavioral weight loss intervention [87]. These evidences, together with our findings, allay the fear that nut intake may promote overweight or obesity.

To the best of knowledge, this is the first meta-analysis examining the effects of nut intake in risk reduction of Mets and overweight/obesity. Nevertheless, the limitations of our study should not be ignored. First of all, the recall and selection bias cannot be avoided in the analyses of Mets and overweight/obesity due to the observational nature of the original studies. Second, heterogeneity was present among the randomized feeding trials, which may influence the pooled estimates of body weight parameters. Third, owing to the lack of data, we cannot conduct stratified analyses for the risk estimates by some important factors (e.g., nut subtype, age and sex). Fourth, due to the quite limited number of cohort studies, the potential of a non-linear association between nut consumption and risk of Mets or overweight/obesity cannot be evaluated. Last but not the least, this meta-analysis has geographical restrictions, as the majority of the included studies were from North America or Europe. Thus, generalization of our finding needs to be considered with caution.

## Conclusion

In summary, the results from this meta-analysis suggest that nut intake may be associated with decreased risks of Mets and overweight/obesity and lower body weight measures. The result supports the current recommendation of nut intake for the prevention of chronic diseases. Future prospective studies are still warranted to enhance our findings and to determine the health benefits of specific nuts.

## Additional files

Additional file 1: **Table S1.** Detailed search strategies. **Table S2.** Excluded articles with reasons at the stage of eligibility. **Table S3.** Baseline characteristics of feeding trials**. Figure S1.** Body weight changes from the individual studies in this meta-analysis. **Figure S2.** Body mass index changes from the individual studies in this meta-analysis. **Figure S3.** Waist circumference changes from the individual studies in this meta-analysis. (DOCX 1311 kb)

## Abbreviations

BMI: Body mass index
CIs: Confidence intervals
Mets: Metabolic syndrome
RRs: Relative risks
WMDs: Weighted mean differences

## References

[1] Alberti KG, Eckel RH, Grundy SM, Zimmet PZ, Cleeman JI, Donato KA (2009). Harmonizing the metabolic syndrome: a joint interim statement of the international diabetes federation task force on epidemiology and prevention; National Heart, Lung, and Blood Institute; American Heart Association; world heart federation; international atherosclerosis society; and International Association for the Study of obesity. Circulation.

[2] Grundy SM (2008). Metabolic syndrome pandemic. Arterioscler Thromb Vasc Biol.

[3] Aguilar M, Bhuket T, Torres S, Liu B, Wong RJ (2015). Prevalence of the metabolic syndrome in the United States, 2003-2012. JAMA.

[4] O'Neill S, O'Driscoll L (2015). Metabolic syndrome: a closer look at the growing epidemic and its associated pathologies. Obes Rev.

[5] Mendonça FM, de Sousa FR, Barbosa AL, Martins SC, Araújo RL, Soares R (2015). Metabolic syndrome and risk of cancer: which link?. Metabolism.

[6] World Health Organization. Fact sheet: obesity and overweight. http://www.who.int/mediacentre/factsheets/fs311/en. Accessed 26 Nov 2017.

[7] Tian Y, Su L, Wang J, Duan X, Jiang X. Fruit and vegetable consumption and risk of the metabolic syndrome: a meta-analysis. Public Health Nutr. 2017; 10.1017/S136898001700310X. 10.1017/S136898001700310X PMC10260986 29151369

[8] Buijsse B, Feskens EJ, Schulze MB, Forouhi NG, Wareham NJ, Sharp S (2009). Fruit and vegetable intakes and subsequent changes in body weight in European populations: results from the project on diet, obesity, and genes (DiOGenes). Am J Clin Nutr.

[9] Lee KW, Cho W (2017). The consumption of dairy products is associated with reduced risks of obesity and metabolic syndrome in Korean women but not in men. Nutrients.

[10] Nordestgaard AT, Thomsen M, Nordestgaard BG (2015). Coffee intake and risk of obesity, metabolic syndrome and type 2 diabetes: a Mendelian randomization study. Int J Epidemiol.

[11] Cocate PG, Natali AJ, de Oliveira A, Alfenas Rde C, Peluzio Mdo C, Longo GZ (2015). Red but not white meat consumption is associated with metabolic syndrome, insulin resistance and lipid peroxidation in Brazilian middle-aged men. Eur J Prev Cardiol.

[12] Rouhani MH, Salehi-Abargouei A, Surkan PJ, Azadbakht L (2014). Is there a relationship between red or processed meat intake and obesity? A systematic review and meta-analysis of observational studies. Obes Rev.

[13] Grosso G, Estruch R (2016). Nut consumption and age-related disease. Maturitas.

[14] Aune D, Keum N, Giovannucci E, Fadnes LT, Boffetta P, Greenwood DC (2016). Nut consumption and risk of cardiovascular disease, total cancer, all-cause and cause-specific mortality: a systematic review and dose-response meta-analysis of prospective studies. BMC Med.

[15] Li Z, Song R, Nguyen C, Zerlin A, Karp H, Naowamondhol K (2010). Pistachio nuts reduce triglycerides and body weight by comparison to refined carbohydrate snack in obese subjects on a 12-week weight loss program. J Am Coll Nutr.

[16] Casas-Agustench P, López-Uriarte P, Bulló M, Ros E, Cabré-Vila JJ, Salas-Salvadó J (2011). Effects of one serving of mixed nuts on serum lipids, insulin resistance and inflammatory markers in patients with the metabolic syndrome. Nutr Metab Cardiovasc Dis.

[17] Bes-Rastrollo M, Wedick NM, Martinez-Gonzalez MA, Li TY, Sampson L, Hu FB (2009). Prospective study of nut consumption, long-term weight change, and obesity risk in women. Am J Clin Nutr.

[18] Fernández-Montero A, Bes-Rastrollo M, Beunza JJ, Barrio-Lopez MT, de la Fuente-Arrillaga C, Moreno-Galarraga L (2013). Nut consumption and incidence of metabolic syndrome after 6-year follow-up: the SUN (Seguimiento Universidad de Navarra, University of Navarra follow-up) cohort. Public Health Nutr.

[19] Freisling H, Noh H, Slimani N, Chajès V, May AM, Peeters PH, et al. Nut intake and 5-year changes in body weight and obesity risk in adults: results from the EPIC-PANACEA study. Eur J Nutr. 2017; 10.1007/s00394-017-1513-0. 10.1007/s00394-017-1513-0 28733927

[20] Hosseinpour-Niazi S, Hosseini S, Mirmiran P, Azizi F (2017). Prospective study of nut consumption and incidence of metabolic syndrome: Tehran lipid and glucose study. Nutrients.

[21] Bes-Rastrollo M, Sabaté J, Gómez-Gracia E, Alonso A, Martínez JA, Martínez-González MA (2007). Nut consumption and weight gain in a Mediterranean cohort: the SUN study. Obesity (Silver Spring).

[22] Lutsey PL, Steffen LM, Stevens J (2008). Dietary intake and the development of the metabolic syndrome: the atherosclerosis risk in communities study. Circulation.

[23] Stroup DF, Berlin JA, Morton SC, Olkin I, Williamson GD, Rennie D (2000). Meta-analysis of observational studies in epidemiology: a proposal for reporting. Meta-analysis of observational studies in epidemiology (MOOSE) group. JAMA.

[24] Wells GA, Shea B, O’Connell D, Peterson J, Welch V, Losos M, et al. The Newcastle-Ottawa Scale (NOS) for Assessing the Quality of Nonrandomised Studies in Meta-Analyses http://www.ohri.ca/programs/clinical_epidemiology/oxford.asp. Accessed 15 Nov 2017.

[25] Schulz KF, Altman DG, Moher D, CONSORT Group (2010). CONSORT 2010 statement: updated guidelines for reporting parallel group randomised trials. BMJ.

[26] Greenland S, Longnecker MP (1992). Methods for trend estimation from summarized dose-response data, with applications to meta-analysis. Am J Epidemiol.

[27] Orsini N, Bellocco R, Greenland S (2006). Generalized least squares for trend estimation of summarized dose-response data. Stata J.

[28] Altman DG, Bland JM (2003). Interaction revisited: the difference between two estimates. BMJ.

[29] Jenkins DJ, Popovich DG, Kendall CW, Vidgen E, Tariq N, Ransom TP (1997). Effect of a diet high in vegetables, fruit, and nuts on serum lipids. Metabolism.

[30] O'Byrne DJ, Knauft DA, Shireman RB (1997). Low fat-monounsaturated rich diets containing high-oleic peanuts improve serum lipoprotein profiles. Lipids.

[31] Spiller GA, Jenkins DA, Bosello O, Gates JE, Cragen LN, Bruce B (1998). Nuts and plasma lipids: an almond-based diet lowers LDL-C while preserving HDL-C. J Am Coll Nutr.

[32] Morgan WA, Clayshulte B (2000). Pecans lower low-density lipoprotein cholesterol in people with normal lipid levels. J Am Diet Assoc.

[33] Zambón D, Sabaté J, Muñoz S, Campero B, Casals E, Merlos M (2000). Substituting walnuts for monounsaturated fat improves the serum lipid profile of hypercholesterolemic men and women. A randomized crossover trial. Ann Intern Med.

[34] Jenkins DJ, Kendall CW, Marchie A, Parker TL, Connelly PW, Qian W (2002). Dose response of almonds on coronary heart disease risk factors: blood lipids, oxidized low-density lipoproteins, lipoprotein(a), homocysteine, and pulmonary nitric oxide: a randomized, controlled, crossover trial. A randomized crossover trial. Circulation.

[35] Sabaté J, Haddad E, Tanzman JS, Jambazian P, Rajaram S (2003). Serum lipid response to the graduated enrichment of a step I diet with almonds: a randomized feeding trial. Am J Clin Nutr.

[36] Wien MA, Sabaté JM, Iklé DN, Cole SE, Kandeel FR (2003). Almonds vs complex carbohydrates in a weight reduction program. Int J Obes Relat Metab Disord.

[37] Lamarche B, Desroches S, Jenkins DJ, Kendall CW, Marchie A, Faulkner D (2004). Combined effects of a dietary portfolio of plant sterols, vegetable protein, viscous fibre and almonds on LDL particle size. Br J Nutr.

[38] Ros E, Núñez I, Pérez-Heras A, Serra M, Gilabert R, Casals E (2004). A walnut diet improves endothelial function in hypercholesterolemic subjects: a randomized crossover trial. Circulation.

[39] Tapsell LC, Gillen LJ, Patch CS, Batterham M, Owen A, Baré M (2004). Including walnuts in a low-fat/modified-fat diet improves HDL cholesterol-to-total cholesterol ratios in patients with type 2 diabetes. Diabetes Care.

[40] Chisholm A, Mc Auley K, Mann J, Williams S, Skeaff M (2005). Cholesterol lowering effects of nuts compared with a canola oil enriched cereal of similar fat composition. Nutr Metab Cardiovasc Dis.

[41] Sabaté J, Cordero-Macintyre Z, Siapco G, Torabian S, Haddad E (2005). Does regular walnut consumption lead to weight gain?. Br J Nutr.

[42] Kocyigit A, Koylu AA, Keles H (2006). Effects of pistachio nuts consumption on plasma lipid profile and oxidative status in healthy volunteers. Nutr Metab Cardiovasc Dis.

[43] Schutte AE, Van Rooyen JM, Huisman HW, Mukuddem-Petersen J, Oosthuizen W, Hanekom SM (2006). Modulation of baroreflex sensitivity by walnuts versus cashew nuts in subjects with metabolic syndrome. Am J Hypertens.

[44] Canales A, Benedí J, Nus M, Librelotto J, Sánchez-Montero JM, Sánchez-Muniz FJ (2007). Effect of walnut-enriched restructured meat in the antioxidant status of overweight/obese senior subjects with at least one extra CHD-risk factor. J Am Coll Nutr.

[45] Hollis J, Mattes R (2007). Effect of chronic consumption of almonds on body weight in healthy humans. Br J Nutr.

[46] Mercanligil SM, Arslan P, Alasalvar C, Okut E, Akgül E, Pinar A (2007). Effect of chronic consumption of almonds on body weight in healthy humans. Eur J Clin Nutr.

[47] Mukuddem-Petersen J, Stonehouse Oosthuizen W, Jerling JC, Hanekom SM, White Z (2007). Effects of a high walnut and high cashew nut diet on selected markers of the metabolic syndrome: a controlled feeding trial. Br J Nutr.

[48] Sheridan MJ, Cooper JN, Erario M, Cheifetz CE (2007). Pistachio nut consumption and serum lipid levels. J Am Coll Nutr.

[49] Gebauer SK, West SG, Kay CD, Alaupovic P, Bagshaw D, Kris-Etherton PM (2008). Effects of pistachios on cardiovascular disease risk factors and potential mechanisms of action: a dose-response study. Am J Clin Nutr.

[50] Olmedilla-Alonso B, Granado-Lorencio F, Herrero-Barbudo C, Blanco-Navarro I, Blázquez-García S, Pérez-Sacristán B (2008). Consumption of restructured meat products with added walnuts has a cholesterol-lowering effect in subjects at high cardiovascular risk: a randomised, crossover, placebo-controlled study. J Am Coll Nutr.

[51] Spaccarotella KJ, Kris-Etherton PM, Stone WL, Bagshaw DM, Fishell VK, West SG (2008). The effect of walnut intake on factors related to prostate and vascular health in older men. Nutr J.

[52] Tapsell LC, Batterham MJ, Teuss G, Tan SY, Dalton S, Quick CJ (2009). Long-term effects of increased dietary polyunsaturated fat from walnuts on metabolic parameters in type II diabetes. Eur J Clin Nutr.

[53] Ma Y, Njike VY, Millet J, Dutta S, Doughty K, Treu JA (2010). Effects of walnut consumption on endothelial function in type 2 diabetic subjects: a randomized controlled crossover trial. Diabetes Care.

[54] Wien M, Bleich D, Raghuwanshi M, Gould-Forgerite S, Gomes J, Monahan-Couch L (2010). Almond consumption and cardiovascular risk factors in adults with prediabetes. J Am Coll Nutr.

[55] Wu H, Pan A, Yu Z, Qi Q, Lu L, Zhang G (2010). Lifestyle counseling and supplementation with flaxseed or walnuts influence the management of metabolic syndrome. J Nutr.

[56] Jaceldo-Siegl K, Sabaté J, Batech M, Fraser GE (2011). Influence of body mass index and serum lipids on the cholesterol-lowering effects of almonds in free-living individuals. Nutr Metab Cardiovasc Dis.

[57] Li SC, Liu YH, Liu JF, Chang WH, Chen CM, Chen CY (2011). Almond consumption improved glycemic control and lipid profiles in patients with type 2 diabetes mellitus. Metabolism.

[58] Foster GD, Shantz KL, Vander Veur SS, Oliver TL, Lent MR, Virus A (2012). A randomized trial of the effects of an almond-enriched, hypocaloric diet in the treatment of obesity. Am J Clin Nutr.

[59] Katz DL, Davidhi A, Ma Y, Kavak Y, Bifulco L, Njike VY (2012). Effects of walnuts on endothelial function in overweight adults with visceral obesity: a randomized, controlled, crossover trial. J Am Coll Nutr.

[60] Damavandi RD, Eghtesadi S, Shidfar F, Heydari I, Foroushani AR (2013). Effects of hazelnuts consumption on fasting blood sugar and lipoproteins in patients with type 2 diabetes. J Res Med Sci.

[61] Orem A, Yucesan FB, Orem C, Akcan B, Kural BV, Alasalvar C (2013). Hazelnut-enriched diet improves cardiovascular risk biomarkers beyond a lipid-lowering effect in hypercholesterolemic subjects. J Clin Lipidol.

[62] Tan SY, Mattes RD (2013). Appetitive, dietary and health effects of almonds consumed with meals or as snacks: a randomized, controlled trial. Eur J Clin Nutr.

[63] Tey SL, Gray AR, Chisholm AW, Delahunty CM, Brown RC (2013). The dose of hazelnuts influences acceptance and diet quality but not inflammatory markers and body composition in overweight and obese individuals. J Nutr.

[64] Abazarfard Z, Salehi M, Keshavarzi S (2014). The effect of almonds on anthropometric measurements and lipid profile in overweight and obese females in a weight reduction program: A randomized controlled clinical trial. J Res Med Sci.

[65] Alves RD, Moreira AP, Macedo VS, de Cássia Gonçalves Alfenas R, Bressan J, Mattes R (2014). Regular intake of high-oleic peanuts improves fat oxidation and body composition in overweight/obese men pursuing a energy-restricted diet. Obesity (Silver Spring).

[66] Babio N, Toledo E, Estruch R, Ros E, Martínez-González MA, Castañer O (2014). Mediterranean diets and metabolic syndrome status in the PREDIMED randomized trial. CMAJ.

[67] Bento AP, Cominetti C, Simões Filho A, Naves MM (2014). Baru almond improves lipid profile in mildly hypercholesterolemic subjects: a randomized, controlled, crossover study. Nutr Metab Cardiovasc Dis.

[68] Gulati S, Misra A, Pandey RM, Bhatt SP, Saluja S (2014). Effects of pistachio nuts on body composition, metabolic, inflammatory and oxidative stress parameters in Asian Indians with metabolic syndrome: a 24-wk, randomized control trial. Nutrition.

[69] Hernández-Alonso P, Salas-Salvadó J, Baldrich-Mora M, Juanola-Falgarona M, Bulló M (2014). Beneficial effect of pistachio consumption on glucose metabolism, insulin resistance, inflammation, and related metabolic risk markers: a randomized clinical trial. Diabetes Care.

[70] Lee YJ, Nam GE, Seo JA, Yoon T, Seo I, Lee JH (2014). Nut consumption has favorable effects on lipid profiles of Korean women with metabolic syndrome. Nutr Res.

[71] Parham M, Heidari S, Khorramirad A, Hozoori M, Hosseinzadeh F, Bakhtyari L (2014). Effects of pistachio nut supplementation on blood glucose in patients with type 2 diabetes: a randomized crossover trial. Rev Diabet Stud.

[72] Wien M, Oda K, Sabaté J (2014). A randomized controlled trial to evaluate the effect of incorporating peanuts into an American Diabetes Association meal plan on the nutrient profile of the total diet and cardiometabolic parameters of adults with type 2 diabetes. Nutr J.

[73] Barbour JA, Howe PR, Buckley JD, Bryan J, Coates AM (2015). Effect of 12 weeks high oleic peanut consumption on cardio-metabolic risk factors and body composition. Nutrients.

[74] Berryman CE, West SG, Fleming JA, Bordi PL, Kris-Etherton PM (2015). Effects of daily almond consumption on cardiometabolic risk and abdominal adiposity in healthy adults with elevated LDL-cholesterol: a randomized controlled trial. J Am Heart Assoc.

[75] Jamshed H, Sultan FA, Iqbal R, Gilani AH (2015). Dietary almonds increase serum HDL cholesterol in coronary artery disease patients in a randomized controlled trial. J Nutr.

[76] Kasliwal RR, Bansal M, Mehrotra R, Yeptho KP, Trehan N (2015). Effect of pistachio nut consumption on endothelial function and arterial stiffness. Nutrition.

[77] Njike VY, Ayettey R, Petraro P, Treu JA, Katz DL (2015). Walnut ingestion in adults at risk for diabetes: effects on body composition, diet quality, and cardiac risk measures. BMJ Open Diabetes Res Care.

[78] Ruisinger JF, Gibson CA, Backes JM, Smith BK, Sullivan DK, Moriarty PM (2015). Statins and almonds to lower lipoproteins. J Clin Lipidol..

[79] Sauder KA, McCrea CE, Ulbrecht JS, Kris-Etherton PM, West SG (2015). Effects of pistachios on the lipid/lipoprotein profile, glycemic control, inflammation, and endothelial function in type 2 diabetes: a randomized trial. Metabolism.

[80] Dhillon J, Tan SY, Mattes RD (2016). Almond consumption during energy restriction lowers Truncal fat and blood pressure in compliant overweight or obese adults. J Nutr.

[81] Le T, Flatt SW, Natarajan L, Pakiz B, Quintana EL, Heath DD (2016). Effects of diet composition and insulin resistance status on plasma lipid levels in a weight loss intervention in women. J Am Heart Assoc.

[82] Rock CL, Flatt SW, Pakiz B, Quintana EL, Heath DD, Rana BK (2016). Effects of diet composition on weight loss, metabolic factors and biomarkers in a 1-year weight loss intervention in obese women examined by baseline insulin resistance status. Metabolism.

[83] Chen CM, Liu JF, Li SC, Huang CL, Hsirh AT, Weng S (2017). Almonds ameliorate glycemic control in Chinese patients with better controlled type 2 diabetes: a randomized, crossover, controlled feeding trial. Nutr Metab (Lond).

[84] Jung H, Chen CO, Blumberg JB, Kwak HK. The effect of almonds on vitamin E status and cardiovascular risk factors in Korean adults: a randomized clinical trial. Eur J Nutr. 2017; 10.1007/s00394-017-1480-5. 10.1007/s00394-017-1480-5 PMC6105263 28695324

[85] Liu Y, Hwang HJ, Ryu H, Lee YS, Kim HS, Park H (2017). The effects of daily intake timing of almond on the body composition and blood lipid profile of healthy adults. Nutr Res Pract.

[86] Neale EP, Tapsell LC, Martin A, Batterham MJ, Wibisono C, Probst YC (2017). Impact of providing walnut samples in a lifestyle intervention for weight loss: a secondary analysis of the HealthTrack trial. Food Nutr Res.

[87] Rock CL, Flatt SW, Barkai HS, Pakiz B, Heath DD (2017). Walnut consumption in a weight reduction intervention: effects on body weight, biological measures, blood pressure and satiety. Nutr J.

[88] Tapsell LC, Lonergan M, Batterham MJ, Neale EP, Martin A, Thorne R (2017). Effect of interdisciplinary care on weight loss: a randomised controlled trial. BMJ Open.

[89] Grundy SM, Cleeman JI, Daniels SR, Donato KA, Eckel RH, Franklin BA (2005). Diagnosis and management of the metabolic syndrome: an American Heart Association/National Heart, Lung, and Blood Institute scientific statement. Circulation.

[90] Krauss RM, Eckel RH, Howard B, Appel LJ, Daniels SR, Deckelbaum RJ (2000). AHA dietary guidelines. Revision 2000: a statement for healthcare professionals from the nutrition Committee of the American Heart Association. Circulation.

[91] Kastorini CM, Milionis HJ, Esposito K, Giugliano D, Goudevenos JA, Panagiotakos DB (2011). The effect of Mediterranean diet on metabolic syndrome and its components: a meta-analysis of 50 studies and 534,906 individuals. J Am Coll Cardiol.

[92] Echeverría G, McGee EE, Urquiaga I, Jiménez P, D'Acuña S, Villarroel L (2017). Inverse associations between a locally validated Mediterranean diet index, overweight/obesity, and metabolic syndrome in Chilean adults. Nutrients.

[93] Mohammadifard N, Salehi-Abargouei A, Salas-Salvadó J, Guasch-Ferré M, Humphries K, Sarrafzadegan N (2015). The effect of tree nut, peanut, and soy nut consumption on blood pressure: a systematic review and meta-analysis of randomized controlled clinical trials. Am J Clin Nutr.

[94] Salas-Salvadó J, Guasch-Ferré M, Bulló M, Sabaté J (2014). Nuts in the prevention and treatment of metabolic syndrome. Am J Clin Nutr.

[95] Boulangé CL, Neves AL, Chilloux J, Nicholson JK, Dumas ME (2016). Impact of the gut microbiota on inflammation, obesity, and metabolic disease. Genome Med.

[96] Lamuel-Raventos RM, Onge MS (2017). Prebiotic nut compounds and human microbiota. Crit Rev Food Sci Nutr.

[97] Jackson CL, Hu FB (2014). Long-term associations of nut consumption with body weight and obesity. Am J Clin Nutr.

[98] Vadivel V, Kunyanga CN, Biesalski HK (2012). Health benefits of nut consumption with special reference to body weight control. Nutrition.

[99] Flores-Mateo G, Rojas-Rueda D, Basora J, Ros E, Salas-Salvadó J (2013). Nut intake and adiposity: meta-analysis of clinical trials. Am J Clin Nutr.

