# Supplementary material for: Nut consumption and risk of metabolic syndrome and overweight/obesity: a meta-analysis of prospective cohort studies and randomized trials
Source: Nutr Metab (Lond). 2018 Jun 22;15:46. doi: 10.1186/s12986-018-0282-y (PMC6013998; doi:10.1186/s12986-018-0282-y)

Additional file 1

**Table S1.** Detailed search strategies.......................................................................................................Page 2-3

**Table S2.** Excluded articles with reasons at the stage of eligibility.......................................................Page 4-14

**Table S3.** Baseline characteristics of feeding trials.............................................................................Page 15-19

**Figure S1.** Body weight changes from the individual studies in this meta-analysis.................................Page 20

**Figure S2.** Body mass index changes from the individual studies in this meta-analysis..........................Page 21

**Figure S3.** Waist circumference changes from the individual studies in this meta-analysis....................Page 22

Table S1. Detailed search strategies

Database: PubMed

| **Search line** | **Search term** | **No. of**  **Hits** |
| --- | --- | --- |
| #1 | ((nuts[MeSH Terms]) OR nut) OR nuts | 9366 |
| #2 | (juglans[MeSH Terms]) OR walnut* | 5967 |
| #3 | (arachis[MeSH Terms]) OR peanut* | 12624 |
| #4 | (corylus[MeSH Terms]) OR hazelnut* | 1032 |
| #5 | (prunus dulcis[MeSH Terms]) OR almond* | 3688 |
| #6 | (pistacia[MeSH Terms]) OR pistachio* | 868 |
| #7 | (anacardium[MeSH Terms]) OR cashew* | 726 |
| #8 | (macadamia[MeSH Terms]) OR macadamia* | 177 |
| #9 | (carya[MeSH Terms]) OR pecan* | 652 |
| #10 | (pinus[MeSH Terms]) OR pine nut* | 5433 |
| #11 | ((bertholletia[MeSH Terms]) OR brazil nut*) OR Amazonia | 2766 |
| **#12** | **#1 OR #2 OR #3 OR #4 OR #5 OR #6 OR #7 OR #8 OR #9 OR #10 OR #11** | **38714** |
| #13 | ((metabolic syndrome X[MeSH Terms]) OR metabolic syndrome) OR Mets | 71260 |
| #14 | ((((((((((((((obesity[MeSH Terms]) OR obesity) OR obese) OR adiposity) OR adipose) OR overweight[MeSH Terms]) OR overweight) OR body mass index[MeSH Terms]) OR body mass index) OR BMI) OR body weight[MeSH Terms]) OR body weight) OR waist circumference[MeSH Terms]) OR waist circumference) OR weight gain | 871191 |
| #15 | (((hypertension[MeSH Terms]) OR hypertension) OR blood pressure[MeSH Terms]) OR blood pressure | 862958 |
| #16 | (((((((hypercholesterolemia[MeSH Terms]) OR hypercholesterolemia) OR dyslipidemia[MeSH Terms]) OR dyslipidemia) OR cholesterol[MeSH Terms]) OR cholesterol) OR triglycerides[MeSH Terms]) OR triglycerides | 348311 |
| #17 | ((((diabetes mellitus[MeSH Terms]) OR diabetes) OR glucose[MeSH Terms]) OR glucose) OR glycemia | 962154 |
| **#18** | **#13 OR #14 OR #15 OR #16 OR #17** | **2521885** |
| **#19** | **#12 AND #18** | **4182** |

Database: Embase

| **Search line** | **Search term** | **No. of**  **Hits** |
| --- | --- | --- |
| #1 | 'nut':ab,ti | 6091 |
| #2 | 'nuts':ab,ti | 6476 |
| #3 | 'walnut*':ab,ti | 2339 |
| #4 | 'peanut*':ab,ti | 14262 |
| #5 | 'hazelnut*':ab,ti | 1537 |
| #6 | 'almond*':ab,ti | 2997 |
| #7 | 'pistachio*':ab,ti | 793 |
| #8 | 'cashew*':ab,ti | 1035 |
| #9 | 'macadamia*':ab,ti | 213 |
| #10 | 'pecan*':ab,ti | 426 |
| #11 | 'pine nut*':ab,ti | 215 |
| #12 | 'brazil nut*':ab,ti | 397 |
| **#13** | **#1 OR #2 OR #3 OR #4 OR #5 OR #6 OR #7 OR #8 OR #9 OR #10 OR #11 OR #12** | **29328** |
| #14 | 'metabolic syndrome':ab,ti OR 'mets':ab,ti | 68461 |
| #15 | 'obesity':ab,ti OR 'obese':ab,ti OR 'adiposity':ab,ti OR 'adipose':ab,ti OR 'overweight':ab,ti OR 'weight gain':ab,ti OR 'body weight':ab,ti OR 'body mass index':ab,ti OR 'bmi':ab,ti OR 'waist circumference':ab,ti | 867663 |
| #16 | 'hypertension':ab,ti OR 'blood pressure':ab,ti | 732908 |
| #17 | 'hypercholesterolemia':ab,ti OR 'dyslipidemia':ab,ti OR 'cholesterol':ab,ti OR 'triglycerides':ab,ti | 343339 |
| #18 | ('diabetes':ab,ti OR 'glucose':ab,ti OR 'glycemia':ab,ti) | 1020172 |
| **#19** | **#14 OR #15 OR #16 OR #17 OR #18** | **2367612** |
| **#26** | **#13 AND #19** | **3658** |

**Table S2. Excluded articles with reasons at the stage of eligibility**

| **No.** | **Authors. Title. Journal origin. Reasons for exclusion** |
| --- | --- |
| 1 | Lee et al. Effects of Dark Chocolate and Almonds on Cardiovascular Risk Factors in Overweight and Obese Individuals: A Randomized Controlled-Feeding Trial. *J Am Heart Assoc.* 2017. doi: 10.1161/JAHA.116.005162. No data reported for body weight parameters. |
| 2 | Kim et al. Benefits of Nut Consumption on Insulin Resistance and Cardiovascular Risk Factors: Multiple Potential Mechanisms of Actions. *Nutrients.* 2017. doi: 10.3390/nu9111271. Review. |
| 3 | Guasch-Ferré et al. Nut Consumption and Risk of Cardiovascular Disease. J Am Coll Cardiol. 2017;70(20):2519-2532. The outcome was incidence of cardiovascular disease, not Mets or overweight/obesity. |
| 4 | Brown et al. Associations between Nut Consumption and Health Vary between Omnivores, Vegetarians, and Vegans. *Nutrients*. 2017;9(11): E1219. Cross-sectional study. |
| 5 | Bamberger et al. A Walnut-Enriched Diet Reduces Lipids in Healthy Caucasian Subjects, Independent of Recommended Macronutrient Replacement and Time Point of Consumption: a Prospective, Randomized, Controlled Trial. *Nutrients*. 2017;9(10): E1097. The outcomes were blood lipids, not body weight parameters. |
| 6 | Nasreddine et al. A minimally processed dietary pattern is associated with lower odds of metabolic syndrome among Lebanese adults. *Public Health Nutr*. 2018;21(1):160-171. The exposure was processed dietary pattern, not nut intake. |
| 7 | Sayer et al. Consuming Almonds vs. Isoenergetic Baked Food Does Not Differentially Influence Postprandial Appetite or Neural Reward Responses to Visual Food Stimuli. *Nutrients.* 2017;9(8): E807. The outcomes were postprandial hunger, desire to eat, fullness, and neural responses to visual food stimuli, not changes in body weight measures. |
| 8 | Adamo et al. Effects of hazelnuts and cocoa on vascular reactivity in healthy subjects: a randomised study. Int J Food Sci Nutr. 2018;69(2):223-234. The outcome was vascular reactivity, not body weight measures. |
| 9 | Berryman et al. Inclusion of Almonds in a Cholesterol-Lowering Diet Improves Plasma HDL Subspecies and Cholesterol Efflux to Serum in Normal-Weight Individuals with Elevated LDL Cholesterol. *J Nutr.* 2017;147(8):1517-23. The outcomes were blood lipid profiles, not changes in body weight measures. |
| 10 | Deon et al. Effect of hazelnut on serum lipid profile and fatty acid composition of erythrocyte phospholipids in children and adolescents with primary hyperlipidemia: A randomized controlled trial. *Clin Nutr*. 2017. 10.1016/j.clnu.2017.05.022. A study of children and adolescents. |
| 11 | Hernández-Alonso et al. Effect of pistachio consumption on the modulation of urinary gut microbiota-related metabolites in prediabetic subjects. *J Nutr Biochem*. 2017;45:48-53. The outcomes were gut microbiota-related metabolites, not changes in body weight measures. |
| 12 | Jomaa et al. Household food insecurity is associated with a higher burden of obesity and risk of dietary inadequacies among mothers in Beirut, Lebanon. *BMC Public Health*. 2017;17(1):567. Cross-sectional study. |
| 13 | Liu et al. Acute Peanut Consumption Alters Postprandial Lipids and Vascular Responses in Healthy Overweight or Obese Men. J Nutr. 2017;147(5):835-840. The outcomes were lipids and vascular responses, not changes in body weight measures. |
| 14 | Sterling et al. Longitudinal Analysis of Nut-Inclusive Diets and Body Mass Index Among Overweight and Obese African American Women Living in Rural Alabama and Mississippi, 2011-2013. *Prev Chronic Dis.* 2017;14:E82. Secondary analysis of a cohort study. |
| 15 | Razquin et al. Dietary energy density and body weight changes after 3 years in the PREDIMED study. *Int J Food Sci Nutr*. 2017;68(7):865-872. The exposure was dietary energy density, not nut intake. |
| 16 | Wang et al. Dietary Patterns Are Associated with Metabolic Outcomes among Adult Samoans in a Cross-Sectional Study. *J Nutr*. 2017 Apr;147(4):628-635. The exposure was mixed-modern dietary pattern, not nut intake. |
| 17 | Mah et al. Cashew consumption reduces total and LDL cholesterol: a randomized, crossover, controlled-feeding trial. *Am J Clin Nutr*. 2017;105(5):1070-1078. No clear data about changes in body weight parameters. |
| 18 | Dhillon et al. Effects of almond consumption on the post-lunch dip and long-term cognitive function in energy-restricted overweight and obese adults. *Br J Nutr*. 2017;117(3):395-402. The outcomes were post-lunch dip and cognitive function, not changes in body weight measures. |
| 19 | Gulati et al. Effect of Almond Supplementation on Glycemia and Cardiovascular Risk Factors in Asian Indians in North India with Type 2 Diabetes Mellitus: A 24-Week Study. *Metab Syndr Relat Disord*. 2017;15(2):98-105. The comparison was preintervention. |
| 20 | Shang et al. Dietary protein from different food sources, incident metabolic syndrome and changes in its components: An 11-year longitudinal study in healthy community-dwelling adults. *Clin Nutr*. 2017;36(6):1540-1548. The exposure was dietary protein intake, not nut consumption. |
| 21 | Njike et al. Inclusion of walnut in the diets of adults at risk for type 2 diabetes and their dietary pattern changes: a randomized, controlled, cross-over trial. *BMJ Open Diabetes Res Care*. 2016;4(1):e000293. A study pertained to overlapping populations. |
| 22 | Jamshed et al. Almond supplementation reduces serum uric acid in coronary artery disease patients: a randomized controlled trial. *Nutr J*. 2016;15(1):77. The outcomes were serum uric acid and blood pressure, not body weight parameters. |
| 23 | Sterling et al. Nut Intake Among Overweight/Obese African-American Women in the Rural South. *Am J Health Behav*. 2016;40(5):585-93. No evaluation of body weight parameters. |
| 24 | Asghari et al. Dietary Approaches to Stop Hypertension (DASH) Dietary Pattern Is Associated with Reduced Incidence of Metabolic Syndrome in Children and Adolescents. *J Pediatr*. 2016;174:178-184.e1. The exposure was DASH diet score, with no measurement of nut intake. |
| 25 | Veissi et al. Mediterranean diet and metabolic syndrome prevalence in type 2 diabetes patients in Ahvaz, southwest of Iran. *Diabetes Metab Syndr*. 2016;10(2 Suppl 1):S26-9. The risk of Mets was only reported for consumption of nuts, legumes, and seeds; and no separate results were provided. |
| 26 | Agebratt et al. A Randomized Study of the Effects of Additional Fruit and Nuts Consumption on Hepatic Fat Content, Cardiovascular Risk Factors and Basal Metabolic Rate. *PLoS One*. 2016;11(1):e0147149. The comparison was fruit intake. |
| 27 | Estruch et al. Effect of a high-fat Mediterranean diet on bodyweight and waist circumference: a prespecified secondary outcomes analysis of the PREDIMED randomised controlled trial. *Lancet Diabetes Endocrinol*. 2016;4(8):666-76. A study pertained to overlapping populations. |
| 28 | Diouf et al. Daily consumption of ready-to-use peanut-based therapeutic food increased fat free mass, improved anemic status but has no impact on the zinc status of people living with HIV/AIDS: a randomized controlled trial. *BMC Public Health*. 2016;16:1. The exposure was RUTF, not nut intake. |
| 29 | Grosso et al. Nut consumption and age-related disease. *Maturitas*. 2016;84:11-6. Review. |
| 30 | Njike et al. Snacking, Satiety, and Weight: A Randomized, Controlled Trial. Am J Health Promot. 2017;31(4):296-301. The comparison was conventional snacks. |
| 31 | Brown *et al*. Association of Nut Consumption with Cardiometabolic Risk Factors in the 2008/2009 New Zealand Adult Nutrition Survey. *Nutrients*. 2015;7(9):7523-42. Cross-sectional study. |
| 32 | Tovar et al. A multifunctional diet improves cardiometabolic-related biomarkers independently of weight changes: an 8-week randomized controlled intervention in healthy overweight and obese subjects. *Eur J Nutr*. 2016;55(7):2295-306. The exposure was multifunctional diet, not nut intake. |
| 33 | Zou et al. A comparison study on the prevalence of obesity and its associated factors among city, township and rural area adults in China. *BMJ Open*. 2015;5(7):e008417. Cross-sectional study. |
| 34 | O'Neil et al. Tree Nut consumption is associated with better adiposity measures and cardiovascular and metabolic syndrome health risk factors in U.S. Adults: NHANES 2005-2010. *Nutr J*. 2015;14:64. Cross-sectional study. |
| 35 | Ha et al. The supplementation effects of peanut sprout on reduction of abdominal fat and health indices in overweight and obese women. *Nutr Res Pract*. 2015;9(3):249-55. The exposure was peanut sprout extracts, not nut intake. |
| 36 | Chen et al. Effect of almond consumption on vascular function in patients with coronary artery disease: a randomized, controlled, cross-over trial. *Nutr J*. 2015;14:61. The outcomes were blood lipids, blood pressure and other biochemical biomarkers, not body weight parameters. |
| 37 | Gadgil et al. Dietary patterns are associated with metabolic risk factors in South Asians living in the United States. *J Nutr*. 2015;145(6):1211-7. The exposure was a dietary pattern, not consumption of nuts. |
| 38 | Hernández-Alonso et al. Effect of pistachio consumption on plasma lipoprotein subclasses in pre-diabetic subjects. *Nutr Metab Cardiovasc Dis*. 2015;25(4):396-402. The outcomes were changes in blood lipids, not body weight parameters. |
| 39 | Barnes et al. Snacking behaviors, diet quality, and body mass index in a community sample of working adults. *J Acad Nutr Diet*. 2015;115(7):1117-23. Cross-sectional study. |
| 40 | Satija et al. Dietary patterns in India and their association with obesity and central obesity. *Public Health Nutr*. 2015;18(16):3031-41. The exposures were 3 dietary patterns, with no reports of the outcomes for nut consumption. |
| 41 | Tey et al. Effects of regular consumption of different forms of almonds and hazelnuts on acceptance and blood lipids. *Eur J Nutr*. 2015;54(3):483-7. The comparison was pre-intervention with nuts. |
| 42 | Moreira et al. Effect of a high-fat meal containing conventional or high-oleic peanuts on post-prandial lipopolysaccharide concentrations in overweight/obese men. *J Hum Nutr Diet*. 2016;29(1):95-104. The outcomes were lipopolysaccharide concentrations, not body weight parameters. |
| 43 | Mohammadifard et al. Inverse association between the frequency of nut consumption and obesity among Iranian population: Isfahan Healthy Heart Program. *Eur J Nutr*. 2015;54(6):925-31. Cross-sectional study. |
| 44 | Nishi et al. Effect of almond consumption on the serum fatty acid profile: a dose-response study. *Br J Nutr*. 2014;112(7):1137-46. The outcomes were serum fatty acid profile and coronary heart disease risk. |
| 45 | Steffen et al. A modified Mediterranean diet pattern is related to lower risk of incident metabolic syndrome over 25 years among young adults: the CARDIA Study. *Br J Nutr*. 2014;112(10):1654-61. The exposure was a modified Mediterranean diet score, not nut intake. |
| 46 | Holligan et al. A moderate-fat diet containing pistachios improves emerging markers of cardiometabolic syndrome in healthy adults with elevated LDL levels. Br J Nutr. 2014;112(5):744-52. The outcomes were blood lipids, not body weight parameters. |
| 47 | Sauder et al. Pistachio nut consumption modifies systemic hemodynamics, increases heart rate variability, and reduces ambulatory blood pressure in well-controlled type 2 diabetes: a randomized trial. *J Am Heart Assoc*. 2014;3(4): e000873. A study pertained to overlapping populations. |
| 48 | Duarte et al. High-oleic peanuts increase diet-induced thermogenesis in overweight and obese men. Nutr Hosp. 2014;29(5):1024-32. The outcome was diet-induced thermogenesis, not body weight parameters. |
| 49 | Moreira et al. High-oleic peanuts: new perspective to attenuate glucose homeostasis disruption and inflammation related obesity. *Obesity (Silver Spring)*. 2014;22(9):1981-8. The outcomes were cardiovascular risk factors, with no clear data reported for body weight parameters. |
| 50 | Jackson et al. Long-term associations of nut consumption with body weight and obesity. *Am J Clin Nutr*. 2014;100 Suppl 1:408S-11S. Review. |
| 51 | Salas-Salvadó et al. Nuts in the prevention and treatment of metabolic syndrome. *Am J Clin Nutr*. 2014;100 Suppl 1:399S-407S. Review. |
| 52 | Hosseinpour-Niazi et al. Cereal, fruit and vegetable fibre intake and the risk of the metabolic syndrome: a prospective study in the Tehran Lipid and Glucose Study. *J Hum Nutr Diet*. 2015;28(3):236-45. The exposure was nut fibre intake, not consumption of nuts. |
| 53 | Barak et al. Adherence to the Dietary Approaches to Stop Hypertension (DASH) diet in relation to obesity among Iranian female nurses. *Public Health Nutr*. 2015 Mar;18(4):705-12. The exposure was DASH diet score, not consumption of nuts. |
| 54 | Choudhury et al. An almond-enriched diet increases plasma α-tocopherol and improves vascular function but does not affect oxidative stress markers or lipid levels. *Free Radic Res.* 2014;48(5):599-606. The outcomes were serum α-tocopherol and vascular function. |
| 55 | Jaceldo-Siegl et al. Tree nuts are inversely associated with metabolic syndrome and obesity: the Adventist health study-2. *PLoS One*. 2014;9(1):e85133. Cross-sectional study. |
| 56 | Kendall et al. Acute effects of pistachio consumption on glucose and insulin, satiety hormones and endothelial function in the metabolic syndrome. *Eur J Clin Nutr*. 2014;68(3):370-5. The outcomes were glucose, insulin, satiety hormones and endothelial function, not body weight parameters. |
| 57 | Wu et al. Walnut-enriched diet reduces fasting non-HDL-cholesterol and apolipoprotein B in healthy Caucasian subjects: A randomized controlled cross-over clinical trial. Metabolism. 2014;63(3):382-91. No data reported for body weight parameters. |
| 58 | Jones et al. A randomized trial on the effects of flavorings on the health benefits of daily peanut consumption. *Am J Clin Nutr*. 2014;99(3):490-6. The intervention was added flavoring, not nut intake. |
| 59 | Kranz et al. Nutrient displacement associated with walnut supplementation in men. *J Hum Nutr Diet*. 2014;27 Suppl 2:247-54. The outcomes were nutrients intake, not body weight parameters. |
| 60 | Berryman et al. Acute Consumption of Walnuts and Walnut Components Differentially Affect Postprandial Lipemia, Endothelial Function, Oxidative Stress, and Cholesterol Efflux in Humans with Mild Hypercholesterolemia. *J Nutr*. 2013;143(6):788-794.The outcomes were blood lipids, endothelial function and oxidative stress, but not nut consumption. |
| 61 | Ibarrola-Jurado et al. Cross-sectional assessment of nut consumption and obesity, metabolic syndrome and other cardiometabolic risk factors: the PREDIMED study. *PLoS One*. 2013;8(2):e57367. Cross-sectional study. |
| 62 | Damasceno et al. Mediterranean diet supplemented with nuts reduces waist circumference and shifts lipoprotein subfractions to a less atherogenic pattern in subjects at high cardiovascular risk. *Atherosclerosis*. 2013;230(2):347-53. A study pertained to overlapping populations. |
| 63 | Ni et al. Substance use, gender, socioeconomic status and metabolic syndrome among adults in Taiwan. *Public Health Nurs*. 2013;30(1):18-28. The exposure was betel-nut chewing, not nut consumption. |
| 64 | Bel-Serrat et al. Food consumption and cardiovascular risk factors in European children: the IDEFICS study. *Pediatr Obes*. 2013;8(3):225-36. The outcome was cardiovascular risk score, not incidence of Mets or overweight/obesity. |
| 65 | Reis et al. Acute and second-meal effects of peanuts on glycaemic response and appetite in obese women with high type 2 diabetes risk: a randomised cross-over clinical trial. Br J Nutr. 2013;109(11):2015-23. The outcomes were glycaemic parameters, not changes in body weight. |
| 66 | Johnston et al. The effect of peanut and grain bar preloads on postmeal satiety, glycemia, and weight loss in healthy individuals: an acute and a chronic randomized intervention trial. *Nutr J*. 2013;12:35. The comparison was grain bar. |
| 67 | Askari et al. The relationship between nut consumption and lipid profile among the Iranian adult population; Isfahan Healthy Heart Program. *Eur J Clin Nutr*. 2013;67(4):385-9. The outcome was blood lipid profiles, not body weight parameters. |
| 68 | Martínez-González et al. A 14-item Mediterranean diet assessment tool and obesity indexes among high-risk subjects: the PREDIMED trial. *PLoS One*. 2012;7(8):e43134. The exposure was adherence to Mediterranean diet, not consumption of nuts. |
| 69 | Cominetti et al. Brazilian nut consumption improves selenium status and glutathione peroxidase activity and reduces atherogenic risk in obese women. *Nutr Res*. 2012;32(6):403-7. No comparative group. |
| 70 | Sánchez-Muniz et al. The antioxidant status response to low-fat and walnut paste-enriched meat differs in volunteers at high cardiovascular Risk carrying different PON-1 polymorphisms. *J Am Coll Nutr*. 2012;31(3):194-205. A study pertained to overlapping populations. |
| 71 | Liu et al. The effect of almonds on inflammation and oxidative stress in Chinese patients with type 2 diabetes mellitus: a randomized crossover controlled feeding trial. *Eur J Nutr*. 2013;52(3):927-35. No evaluation of changes in body weight parameters. |
| 72 | West et al. Diets containing pistachios reduce systolic blood pressure and peripheral vascular responses to stress in adults with dyslipidemia. *Hypertension*. 2012;60(1):58-63. No evaluation of body weight parameters. |
| 73 | O'Neil et al. Out-of-hand nut consumption is associated with improved nutrient intake and health risk markers in US children and adults: National Health and Nutrition Examination Survey 1999-2004. *Nutr Res*. 2012;32(3):185-94. Cross-sectional study. |
| 74 | Min et al. Breakfast patterns are associated with metabolic syndrome in Korean adults. *Nutr Res Pract*. 2012;6(1):61-7. The exposures were breakfast patterns, with no reports for nut consumption. |
| 75 | Stockler-Pinto et al. Effect of Brazil nut supplementation on plasma levels of selenium in hemodialysis patients: 12 months of follow-up. *J Ren Nutr*. 2012 Jul;22(4):434-9. No comparative group. |
| 76 | Wang et al. Effects of pistachios on body weight in Chinese subjects with metabolic syndrome. *Nutr J*. 2012;11:20. No clear reports for body weight parameters. |
| 77 | Duffey et al. Dietary patterns matter: diet beverages and cardiometabolic risks in the longitudinal Coronary Artery Risk Development in Young Adults (CARDIA) Study. *Am J Clin Nutr*. 2012;95(4):909-15. The exposures were different dietary patterns, not consumption of nuts. |
| 78 | Vadivel et al. Health benefits of nut consumption with special reference to body weight control. *Nutrition*. 2012;28(11-12):1089-97. Review. |
| 79 | Hosseinpour-Niazi et al. Inverse association between fruit, legume, and cereal fiber and the risk of metabolic syndrome: Tehran Lipid and Glucose Study. *Diabetes Res Clin Pract*. 2011;94(2):276-83. The exposure was nut fibre intake, not nut intake. |
| 80 | O'Neil et al. Nut consumption is associated with decreased health risk factors for cardiovascular disease and metabolic syndrome in U.S. adults: NHANES 1999-2004. *J Am Coll Nutr*. 2011;30(6):502-10. Cross-sectional study. |
| 81 | Aronis et al. Short-term walnut consumption increases circulating total adiponectin and apolipoprotein-A concentrations, but does not affect markers of inflammation or vascular injury in obese humans with the metabolic syndrome: data from a double-blinded, randomized, placebo-controlled study. *Metabolism*. 2012;61(4):577-82. The outcomes were blood lipids and inflammatory markers, not body weight parameters. |
| 82 | Tulipani et al. Metabolomics unveils urinary changes in subjects with metabolic syndrome following 12-week nut consumption. *J Proteome Res*. 2011;10(11):5047-58. The outcomes were metabolomics unveils urinary changes, not body weight parameters. |
| 83 | Davidi et al. The effect of the addition of daily fruit and nut bars to diet on weight, and cardiac risk profile, in overweight adults. *J Hum Nutr Diet*. 2011;24(6):543-51. The exposure was a combination of fruit and nut bars, not nut consumption. |
| 84 | Matthews et al. The risk of child and adolescent overweight is related to types of food consumed. *Nutr J.* 2011;10:71. A study of children or adolescents |
| 85 | Mozaffarian et al. Changes in diet and lifestyle and long-term weight gain in women and men. *N Engl J Med*. 2011;364(25):2392-404. A cohort study evaluating long-term changes in body weight. |
| 86 | Jaceldo-Siegl et al. Influence of body mass index and serum lipids on the cholesterol-lowering effects of almonds in free-living individuals. Nutr Metab Cardiovasc Dis. 2011;21 Suppl 1:S7-13. A study pertained to overlapping populations. |
| 87 | Maranhão et al. Brazil nuts intake improves lipid profile, oxidative stress and microvascular function in obese adolescents: a randomized controlled trial. *Nutr Metab (Lond)*. 2011;8:32. A study of adolescents. |
| 88 | Damasceno et al. Crossover study of diets enriched with virgin olive oil, walnuts or almonds. Effects on lipids and other cardiovascular risk markers. *Nutr Metab Cardiovasc Dis*. 2011;21 Suppl 1:S14-20. The comparison was pre-intervention with nuts. |
| 89 | Kendall et al. The impact of pistachio intake alone or in combination with high-carbohydrate foods on post-prandial glycemia. The outcome was glycemia, not body weight parameters. |
| 90 | Martínez-González et al. Nut consumption, weight gain and obesity: Epidemiological evidence. *Nutr Metab Cardiovasc Dis*. 2011;21 Suppl 1:S40-5. Review. |
| 91 | Kouki et al. Food consumption, nutrient intake and the risk of having metabolic syndrome: the DR's EXTRA Study. *Eur J Clin Nutr*. 2011;65(3):368-77. The risk estimates were reported for consumption of legumes and nuts, and no separate results were provided. |
| 92 | Kalgaonkar et al. Differential effects of walnuts vs almonds on improving metabolic and endocrine parameters in PCOS. *Eur J Clin Nutr*. 2011;65(3):386-93. The comparison was different type of nuts. |
| 93 | Jalali-Khanabadi et al. Effects of almond dietary supplementation on coronary heart disease lipid risk factors and serum lipid oxidation parameters in men with mild hyperlipidemia. *J Altern Complement Med*. 2010;16(12):1279-83. The comparison was preintervention. |
| 94 | Razquin et al. A Mediterranean diet rich in virgin olive oil may reverse the effects of the -174G/C IL6 gene variant on 3-year body weight change. Mol Nutr Food Res. 2010;54 Suppl 1:S75-82. A study pertained to overlapping populations. |
| 95 | Tey et al. Effects of different forms of hazelnuts on blood lipids and α-tocopherol concentrations in mildly hypercholesterolemic individuals. *Eur J Clin Nutr*. 2011;65(1):117-24. The comparison was different forms of hazelnuts. |
| 96 | Amini et al. Relationship between major dietary patterns and metabolic syndrome among individuals with impaired glucose tolerance. *Nutrition*. 2010;26(10):986-92. The exposures were dietary patterns, not the consumption of nuts. |
| 97 | Kendall et al. Nuts, metabolic syndrome and diabetes. *Br J Nutr*. 2010;104(4):465-73. Review. |
| 98 | Casas-Agustench et al. Cross-sectional association of nut intake with adiposity in a Mediterranean population. *Nutr Metab Cardiovasc Dis*. 2011;21(7):518-25. Cross-sectional study. |
| 99 | Pawlak et al. Beliefs, benefits, barriers, attitude, intake and knowledge about peanuts and tree nuts among WIC participants in eastern North Carolina. Nutr Res Pract. 2009;3(3):220-5. Cross-sectional study. |
| 100 | McKiernan et al. Effects of peanut processing on body weight and fasting plasma lipids. Br J Nutr. 2010;104(3):418-26. The comparison was different peanut processing methods. |
| 101 | Torabian et al. Long-term walnut supplementation without dietary advice induces favorable serum lipid changes in free-living individuals. *Eur J Clin Nutr*. 2010;64(3):274-9. The outcomes were improvements in serum lipids, not changes in body weight parameters. |
| 102 | Stockler-Pinto et al. Effect of Brazil nut supplementation on the blood levels of selenium and glutathione peroxidase in hemodialysis patients. *Nutrition*. 2010;26(11-12):1065-9. The outcomes were blood levels of selenium and glutathione peroxidase, not changes in body weight parameters. |
| 103 | López-Uriarte et al. Effect of nut consumption on oxidative stress and the endothelial function in metabolic syndrome. Clin Nutr. 2010;29(3):373-80. A study pertained to overlapping populations. |
| 104 | Brennan et al. Walnut consumption increases satiation but has no effect on insulin resistance or the metabolic profile over a 4-day period. *Obesity (Silver Spring)*. 2010;18(6):1176-82. The outcomes were cardiometabolic parameters, but not body weight parameters. |
| 105 | Jönsson et al. Beneficial effects of a Paleolithic diet on cardiovascular risk factors in type 2 diabetes: a randomized cross-over pilot study. *Cardiovasc Diabetol*. 2009;8:35. The exposure was a paleolithic diet, not nut consumption. |
| 106 | Liu et al. Dietary pattern, the metabolic syndrome, and left ventricular mass and systolic function: the Multi-Ethnic Study of Atherosclerosis. *Am J Clin Nutr*. 2009;90(2):362-8. The exposure was dietary pattern, not nut consumption. |
| 107 | Zaveri et al. The effect of including a conventional snack (cereal bar) and a nonconventional snack (almonds) on hunger, eating frequency, dietary intake and body weight. J Hum Nutr Diet. 2009;22(5):461-8. The comparison was cereal bar. |
| 108 | Deshmukh-Taskar et al. Dietary patterns associated with metabolic syndrome, sociodemographic and lifestyle factors in young adults: the Bogalusa Heart Study. *Public Health Nutr*. 2009;12(12):2493-503. The exposure was Western or prudent dietary pattern, not nut consumption. |
| 109 | Claesson et al. Two weeks of overfeeding with candy, but not peanuts, increases insulin levels and body weight. *Scand J Clin Lab Invest*. 2009;69(5):598-605. The comparison is candy intake. |
| 110 | Strunz et al. Brazil nut ingestion increased plasma selenium but had minimal effects on lipids, apolipoproteins, and high-density lipoprotein function in human subjects. Nutr Res. 2008;28(3):151-5. No comparative group. |
| 111 | Mattes et al. Impact of peanuts and tree nuts on body weight and healthy weight loss in adults. J Nutr. 2008;138(9):1741S-1745S. Review. |
| 112 | Salas-Salvadó et al. Effect of a Mediterranean diet supplemented with nuts on metabolic syndrome status: one-year results of the PREDIMED randomized trial. *Arch Intern Med*. 2008;168(22):2449-58. A study pertained to overlapping populations. |
| 113 | Mattes et al. Impact of peanuts and tree nuts on body weight and healthy weight loss in adults. *J Nutr*. 2008;138(9):1741S-1745S. Review. |
| 114 | Jenkins et al. Almonds reduce biomarkers of lipid peroxidation in older hyperlipidemic subjects. *J Nutr*. 2008;138(5):908-13. A study pertained to overlapping populations. |
| 115 | Sánchez-Taínta et al. Adherence to a Mediterranean-type diet and reduced prevalence of clustered cardiovascular risk factors in a cohort of 3,204 high-risk patients. *Eur J Cardiovasc Prev Rehabil*. 2008;15(5):589-93. The exposure was a Mediterranean diet score, not nut consumption. |
| 116 | Sabaté et al. Nut consumption and change in weight: the weight of the evidence. *Br J Nutr*. 2007 ;98(3):456-7. Comment. |
| 117 | Davis et al. The effects of high walnut and cashew nut diets on the antioxidant status of subjects with metabolic syndrome. *Eur J Nutr*. 2007;46(3):155-64. The outcomes were antioxidant status, not changes in body weight parameters. |
| 118 | Estruch et al. Effects of a Mediterranean-style diet on cardiovascular risk factors: a randomized trial. *Ann Intern Med*. 2006;145(1):1-11. A study pertained to overlapping populations. |
| 119 | Sánchez-Villegas et al. Adherence to a Mediterranean dietary pattern and weight gain in a follow-up study: the SUN cohort. *Int J Obes (Lond)*. 2006;30(2):350-8. The exposure was a Mediterranean dietary pattern, not nut intake. |
| 120 | Hiraoka-Yamamoto et al. Serum lipid effects of a monounsaturated (palmitoleic) fatty acid-rich diet based on macadamia nuts in healthy, young Japanese women. *Clin Exp Pharmacol Physiol*. 2004;31 Suppl 2:S37-8. The comparison was coconuts and butter, not nut-free diet. |
| 121 | Schröder et al. Adherence to the traditional mediterranean diet is inversely associated with body mass index and obesity in a spanish population. *J Nutr*. 2004;134(12):3355-61. The exposure was a mediterranean diet score, not nut intake. |
| 122 | Sabaté et al. Nut consumption and body weight. *Am J Clin Nutr*. 2003;78(3 Suppl):647S-650S. Review. |
| 123 | Jenkins et al. The effect of combining plant sterols, soy protein, viscous fibers, and almonds in treating hypercholesterolemia. *Metabolism*. 2003;52(11):1478-83. The intervention was a portfolio diet containing soy foods, viscous fibers, plant sterols, and almonds. |
| 124 | Garcı´a-Lorda et al. Nut consumption, body weight and insulin resistance. *Eur J Clin Nutr*. 2003;57:8S-11S. Review. |
| 125 | Morgan et al. Effects of walnut consumption as part of a low-fat, low-cholesterol diet on serum cardiovascular risk factors. *Int J Vitam Nutr Res*. 2002;72(5):341-7. The outcomes were measurements of cardiovascular risk factors, without evaluation of body weight parameters. |
| 126 | Lovejoy et al. Effect of diets enriched in almonds on insulin action and serum lipids in adults with normal glucose tolerance or type 2 diabetes. *Am J Clin Nutr*. 2002;76(5):1000-6. The outcomes were serum lipids and insulin sensitivity, without evaluation of body weight parameters. |
| 127 | Fraser et al. Effect on body weight of a free 76 Kilojoule (320 calorie) daily supplement of almonds for six months. *J Am Coll Nutr*. 2002;21(3):275-83. No comparative group |
| 128 | Jiang. Nut and Peanut Butter Consumption and Risk of Type 2 Diabetes in Women. *JAMA*. 2002;288(20):2554-60. Cohort study evaluating weight gain. |
| 129 | Alper et al. Effects of chronic peanut consumption on energy balance and hedonics. *Int J Obes Relat Metab Disord*. 2002;26(8):1129-37. The comparison was peanut supplementation with no guidance. |
| 130 | Almario et al. Effects of walnut consumption on plasma fatty acids and lipoproteins in combined hyperlipidemia. *Am J Clin Nutr*. 2001;74(1):72-9. No clear report of body weight parameters. |
| 131 | Edwards et al. Effect of pistachio nuts on serum lipid levels in patients with moderate hypercholesterolemia. *J Am Coll Nutr*. 1999;18(3):229-32. The outcomes were serum lipid changes, not the incidence of Mets or overweight/obesity. |
| 132 | Sabaté et al. Effects of walnuts on serum lipid levels and blood pressure in normal men. *N Engl J Med*. 1993;328(9):603-7. The outcomes were serum lipid levels and blood pressure, not the incidence of Mets or overweight/obesity. |

**Table S3.** Baseline characteristics of randomized feeding trials

| **Study, Country** | **Year** | **Design** | **Nut-enriched**  **diet** | **Control diet** | **N** | **Age**  **(yr)** | **Duration**  **(wk)** | **CONSORT score** |
| --- | --- | --- | --- | --- | --- | --- | --- | --- |
| Jenkins, Canada [29] | 1997 | Randomized, crossover | Mixed nuts, 60-120 g/d | Habitual diet | 10 | 33 | 2 | 16 |
| O'Byrne, US [30] | 1997 | Single-arm trial with a control group | Peanuts, 35-68 g/d | Low-fat diet | 25 | 50-65 | 24 | 10 |
| Spiller, US [31] | 1998 | Randomized, controlled, parallel | Supply of almonds, 100 g/d | Cheddar cheese, butter, and bye crackers | 30 | 53 | 4 | 13 |
| Morgan, US [32] | 2000 | Randomized, controlled, parallel | Supply of pecans, 68 g/d | Habitual diet | 19 | 45 | 8 | 17 |
| Zambón, Spain [33] | 2000 | Randomized, crossover | Walnuts partially replaced monounsaturated fat, 41-56 g/d | Mediterranean diet | 49 | 56 | 6 | 10 |
| Jenkins, Canada [34] | 2002 | Randomized, crossover | Supply of almonds, (73±3) g/d | NCEP Step II | 25 | 64 | 4 | 10 |
| Sabaté, US [35] | 2003 | Randomized, crossover | Almonds replaced 20% of energy in the control diet, 68 g/d | NCEP Step I | 25 | 41 | 4 | 14 |
| Wien, US [36] | 2003 | Randomized, controlled, parallel | Unblanched almonds, 84 g/d | Low-fat diet | 65 | 55 | 24 | 18 |
| Lamarche, Canada [37] | 2004 | Randomized, crossover | Almonds, 2.9 g/4.2 MJ | Low-fat diet | 12 | 65 | 4 | 12 |
| Ros, Spain [38] | 2004 | Randomized, crossover | Walnuts, 40-65 g/d | Mediterranean diet | 21 | 55 | 4 | 14 |
| Tapsell, Australia [39] | 2004 | Randomized, controlled, parallel | Supply of walnuts, 30 g/d | Low-fat diet | 58 | 59 | 24 | 15 |
| Chisholm,  New Zealand [40] | 2005 | Randomized, crossover | Supply of mixed nuts, 1 serving/d | Habitual diet plus cereal | 28 | 48 | 6 | 14 |
| Sabaté, US [41] | 2005 | Randomized, crossover | Supply of walnuts, 28 g/d | Habitual diet | 90 | 55 | 24 | 12 |
| Kocyigit, Turkey [42] | 2006 | Randomized, controlled, parallel | Pistachios, 65-75 g/d | Habitual diet | 44 | 33 | 3 | 13 |
| Schutte,  South Africa [43] | 2006 | Randomized, controlled, parallel | Walnuts or cashews, 63-108 g/d | Habitual diet | 62 | 45 | 8 | 8 |
| Canales, Spain [44] | 2007 | Randomized, crossover | Walnut-enriched restructured meat, 150 g/week | Habitual diet plus meat, without walnut | 22 | 54 | 5 | 10 |
| Hollis, US0 [45] | 2007 | Randomized, crossover | Almonds, 1440 kJ/d | Habitual Diet | 24 | 24 | 10 | 12 |
| Mercanligil, Turkey [46] | 2007 | Randomized, crossover | Hazalnuts, 40g/d | Low-fat and -cholesterol,  high-carbohydrate diet | 15 | 48 | 8 | 12 |
| Mukuddem-Petersen, South Africa [47] | 2007 | Randomized, controlled, parallel | Walnuts or cashews, 20% of daily energy | Habitual diet | 64 | 45 | 8 | 11 |
| Sheridan, US [48] | 2007 | Randomized, crossover | Pistachios, 15% of daily energy | Habitual diet | 15 | 60 | 4 | 14 |
| Gebauer, US [49] | 2008 | Randomized, crossover | Pistachios, 15% of daily energy | Low-fat diet | 28 | 48 | 4 | 14 |
| Olmedilla-Alonso,  Spain [50] | 2008 | Randomized, crossover | 20% walnuts added | Habitual diet without walnut | 25 | 54 | 5 | 10 |
| Spaccarotella, US [51] | 2008 | Randomized, crossover | Supply of walnuts, 75 g/d | Habitual diet | 22 | 66 | 8 | 14 |
| Tapsell, Australia [52] | 2009 | Randomized, controlled, parallel | Walnuts, 30 g/d | Low-fat diet | 50 | 54 | 48 | 17 |
| Li, US [15] | 2010 | Randomized, controlled, parallel | Pistachios, 53 g/day | Weight reduction diet plus pretzels | 59 | 46 | 12 | 14 |
| Ma, US [53] | 2010 | Randomized, crossover | Walnuts, 56 g/d | Habitual diet | 24 | 58 | 8 | 18 |
| Wien, US [54] | 2010 | Randomized, controlled, parallel | Almond, 20% of energy in ADA diet | ADA diet | 65 | 54 | 16 | 18 |
| Wu, China [55] | 2010 | Randomized, controlled, parallel | Walnut incorporated into bread, 30 g/d | Diet according to the AHA guidelines | 189 | 48 | 12 | 17 |
| Casas-Agustench,  Spain [16] | 2011 | Randomized, controlled, parallel | Supply of mixed nuts, 30 g/d | Habitual diet | 50 | 52 | 12 | 16 |
| Jaceldo-  Siegl, US [56] | 2011 | Controlled, crossover | Almond, 15% of daily energy | Habitual diet | 81 | 49 | 24 | 11 |
| Li, Taiwan [57] | 2011 | Randomized, crossover | Almonds replaced 20% of daily energy | NCEP Step II | 20 | 58 | 8 | 16 |
| Foster, US [58] | 2012 | Randomized, controlled, parallel | Supply of almond, 28 g/d | Hypocaloric diet | 123 | 47 | 72 | 18 |
| Katz, US [59] | 2012 | Randomized, crossover | Walnuts, 56 g/d | *ad libitum* diet | 46 | 57 | 8 | 17 |
| Damavandi, Iran [60] | 2013 | Randomized, controlled, parallel | Hazelnuts replaced 10% of energy | Habitual diet | 50 | 56 | 8 | 19 |
| Orem, Turkey [61] | 2013 | Randomized, crossover | Hazelnut, 49-86 g/day | Habitual diet | 21 | 45 | 4 | 10 |
| Tan, Australia [62] | 2013 | Randomized, controlled, parallel | Almonds consumed with meals or as snacks, 43 g/d | Habitual diet without nuts and seeds | 137 | 30 | 4 | 18 |
| Tey, New Zealand [63] | 2013 | Randomized, controlled, parallel | Hazelnuts, 30 or 60 g/d | Habitual diet | 107 | 43 | 12 | 19 |
| Abazarfard, Iran [64] | 2014 | Randomized, controlled, parallel | Almond as snacks, 50 g/d | Balanced hypocaloric diet | 108 | 43 | 12 | 18 |
| Alves, Brazil [65] | 2014 | Randomized, controlled, parallel | High-oleic peanut, 50g/d | Nut-free hypocaloric diet | 43 | 28 | 4 | 12 |
| Babio, Spain [66] | 2014 | Randomized, controlled, parallel | Supply of mixed nuts, 30 g/d | Low-fat diet | 3412 | 67 | 336 | 21 |
| Bento, Brazil [67] | 2014 | Randomized, crossover | Supply of almonds, 20 g/d | A corn starch capsule | 20 | 35 | 6 | 14 |
| Gulati, India [68] | 2014 | Randomized, controlled, parallel | Pistachios, 20% of daily energy | Guideline-based diet | 60 | 43 | 24 | 14 |
| Hernández-Alonso,  Spain [69] | 2014 | Randomized, crossover | Pistachios, 57 g/day | Diet with energy adjusted to compensate for the  energy from pistachios | 54 | 55 | 16 | 19 |
| Lee, Korea [70] | 2014 | Randomized, controlled, parallel | Supply of mixed nuts, 30g/d | Prudent diet | 60 | 35-65 | 6 | 18 |
| Parham, Iran [71] | 2014 | Randomized, crossover | Pistachio, 50 g/d | Habitual diet | 48 | 52 | 12 | 16 |
| Wien, US [72] | 2014 | Randomized, controlled, parallel | Peanuts, 20% of energy from ADA diet | ADA diet | 60 | 62 | 24 | 16 |
| Barbour, Australia [73] | 2015 | Randomized, crossover | Peanuts, 15%-20% of daily energy | Habitual diet | 61 | 65 | 12 | 15 |
| Berryman, US [74] | 2015 | Randomized, crossover | Almonds, 1.5 oz/d | Identical diet | 48 | 50 | 6 | 19 |
| Jamshed, Pakistan [75] | 2015 | Randomized, crossover | Almonds, 10 g/d | Habitual diet | 150 | 60 | 12 | 20 |
| Kasliwal, India [76] | 2015 | Randomized, controlled, parallel | Pistachios, 80 g/d | Healthy diet | 42 | 39 | 12 | 13 |
| Njike, US [77] | 2015 | Randomized, crossover | Walnuts, 56 g/d | Habitual diet | 112 | 55 | 24 | 17 |
| Ruisinger, US [78] | 2015 | Randomized, controlled, parallel | Almonds, 100 g/d | ATP-III TLC Diet | 48 | 60 | 4 | 16 |
| Sauder, US [79] | 2015 | Randomized, crossover | Pistachio, 20% of daily energy | Nutritionally-adequate diet | 30 | 56 | 4 | 19 |
| Dhillon, US [80] | 2016 | Randomized, controlled, parallel | Almond, 15% of daily energy | Energy-restricted diet | 86 | 31 | 12 | 14 |
| Le, US [81] | 2016 | Randomized, controlled, parallel | Walnuts, 42 g/d | Hypocaloric diet | 163 | 50 | 24 | 15 |
| Rock, US [82] | 2016 | Randomized, controlled, parallel | Walnuts, 15% of daily energy | Hypocaloric diet | 80 | 50 | 48 | 18 |
| Chen, Taiwan [83] | 2017 | Randomized, crossover | Almonds, 60 g/d | NCEP step II | 40 | 55 | 12 | 17 |
| Jung, Korea [84] | 2017 | Randomized, crossover | Almonds, 56 g/d | Isocaloric cookies | 84 | 52 | 4 | 11 |
| Liu, Korea [85] | 2017 | Randomized, controlled, parallel | Almonds, 56 g/d | Hypercaloric diet | 169 | 26 | 16 | 18 |
| Neale, Australia [86] | 2017 | Randomized, controlled, parallel | Walnuts, 30 g/d | Guideline-based diet | 189 | 43 | 12 | 14 |
| Rock, US [87] | 2017 | Randomized, controlled, parallel | Walnuts, 15% of daily energy | Hypocaloric diet | 100 | 53 | 24 | 16 |
| Tapsell, Australia [88] | 2017 | Randomized, controlled, parallel | Walnuts, 30 g/d | Guideline-based diet | 117 | 45 | 24 | 15 |

ADA, American Diabetes Association; AHA, American Heart Association; ATP, Adult Treatment Panel; CONSORT, CONsolidated Standards Of Reporting Trials; NCEP, National Cholesterol Education Program; TLC, Therapeutic Lifestyle Changes

**Figure S1.** Body weight changes from the individual studies in this meta-analysis
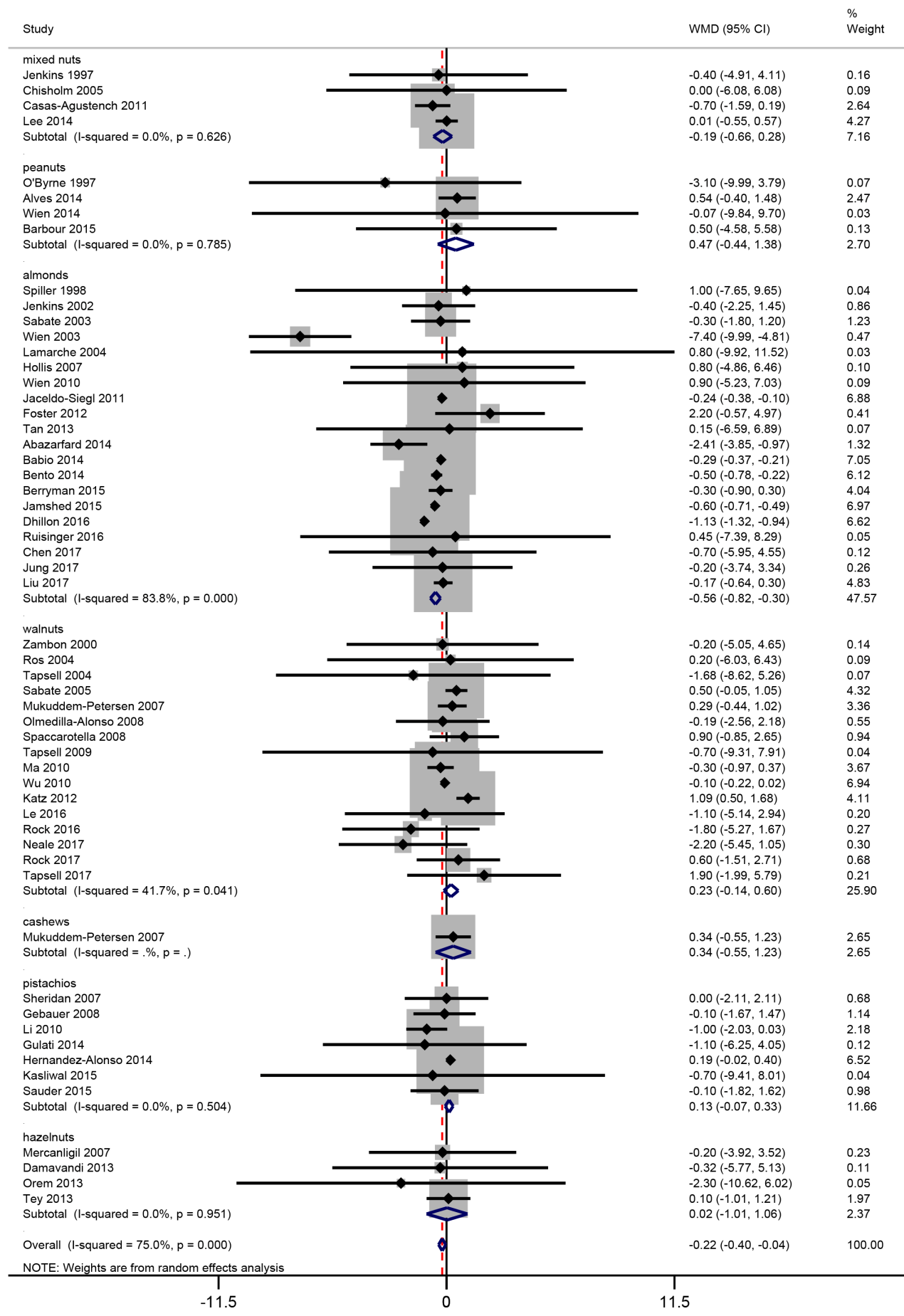


**Figure S2.** Body mass index changes from the individual studies in this meta-analysis
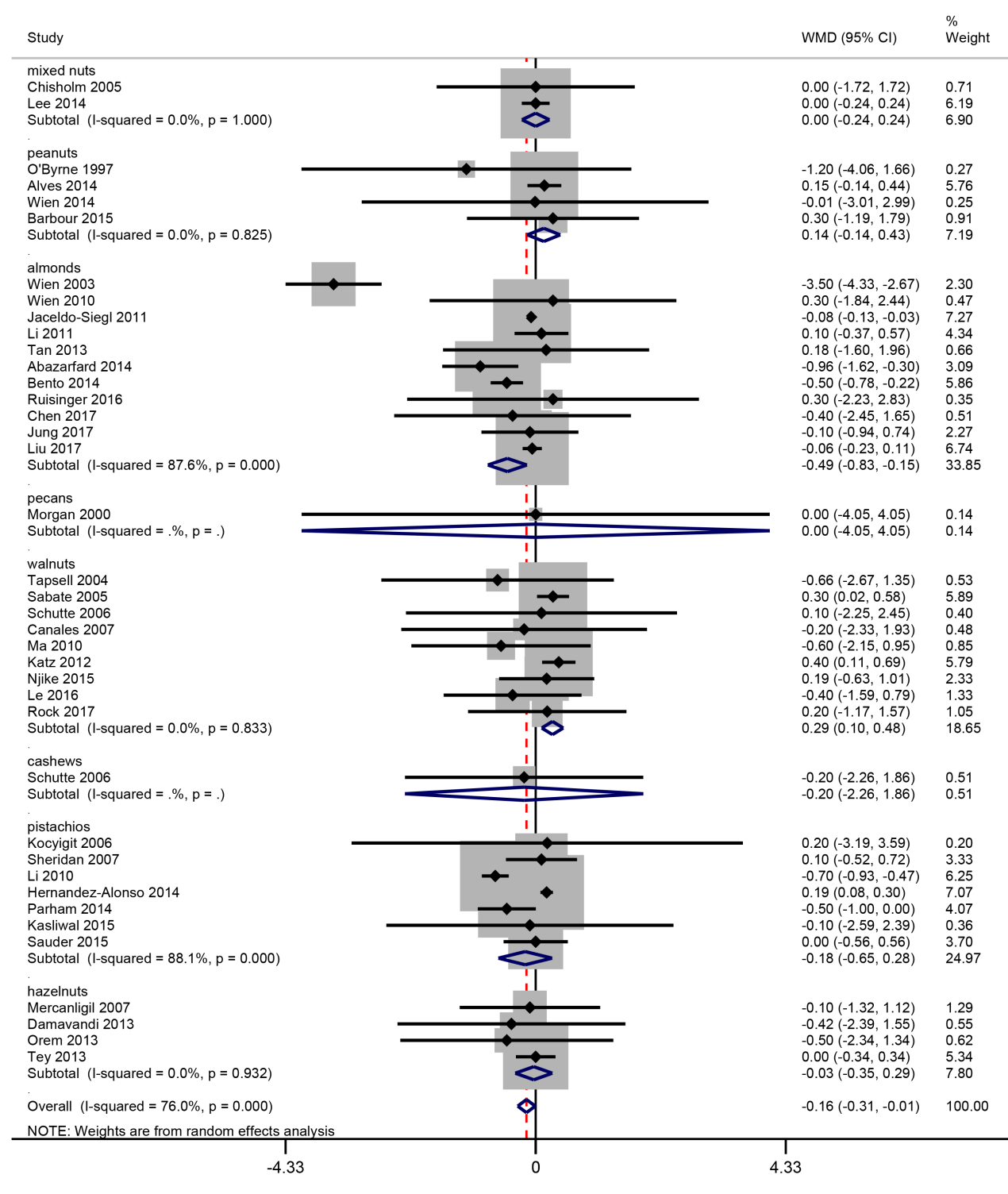


**Figure S3.** Waist circumference changes from the individual studies in this meta-analysis
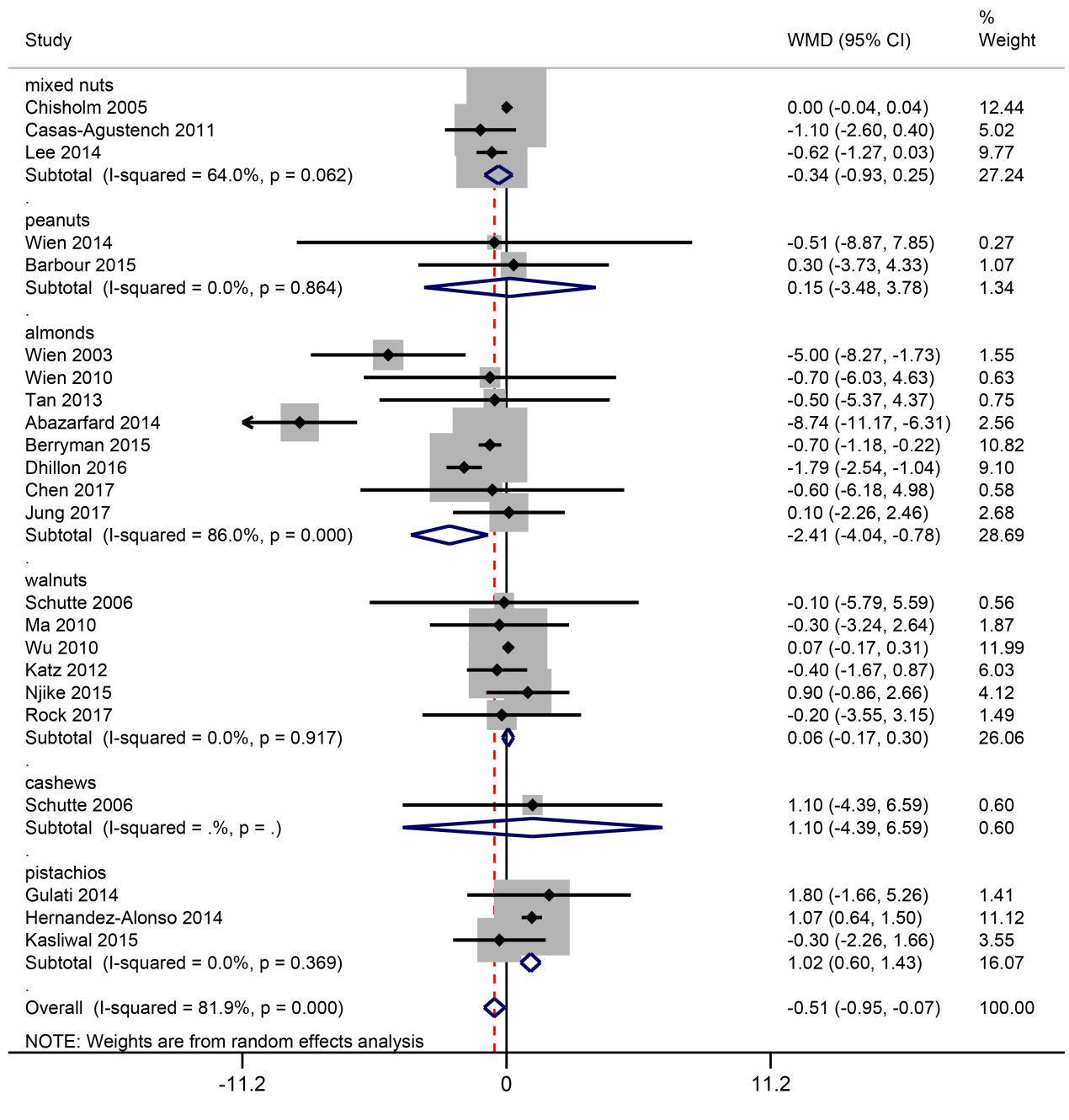

Supplement: Supplementary file 1 — Table S1. Detailed search strategies. Table S2. Excluded articles with reasons at the stage of eligibility. Table S3. Baseline characteristics of feeding trials. Figure S1. Body weight changes from the individual studies in this meta-analysis. Figure S2. Body mass index changes from the individual studies in this meta-analysis. Figure S3. Waist circumference changes from the individual studies in this meta-analysis. (DOCX 1311 kb) [file 12986_2018_282_MOESM1_ESM.docx]
